# Supplementary material for: Co-evolving infectivity and expression patterns drive the diversification of endogenous retroviruses
Source: EMBO J. 2025 Jun 5;45(6):1889–908. doi: 10.1038/s44318-025-00471-8 (PMC12992720; doi:10.1038/s44318-025-00471-8)
Supplement: Supplementary file 2 — Appendix [file 44318_2025_471_MOESM2_ESM.pdf]

Appendix

Co-evolving infectivity and expression patterns drive the diversification of endogenous retrovirus

Kirsten-André Senti[1,*], Baptiste Rafanel[1,2], Dominik Handler[1], Carolin Kosiol[3], Christian Schlötterer[4], Julius Brennecke[1,*]

[1] Institute of Molecular Biotechnology of the Austrian Academy of Sciences (IMBA), Vienna BioCenter (VBC); Dr. Bohr-Gasse 3, 1030 Vienna, Austria
[2] Vienna BioCenter PhD Program, Doctoral School of the University of Vienna and Medical University of Vienna, Vienna, Austria
[3] University of St Andrews, Centre for Biological Diversity; St Andrews, Scotland, UK
[4] Institut für Populationsgenetik, Vetmeduni Vienna; Veterinärplatz 1, 1210 Vienna, Austria
[*] Corresponding authors: senti@imba.oeaw.ac.at, julius.brennecke@imba.oeaw.ac.at

Appendix Figure S1 2
Appendix Figure S2 3
Appendix Figure S3 4
Appendix Figure S4 5
Appendix Figure S5 7
Appendix Figure S6 8
Appendix Figure S7 10
Appendix Figure S8 12
Appendix Figure S9 13
Appendix Figure S10 14
Appendix Figure S11 16
Appendix Figure S12 18
Appendix Figure S13 24
Appendix Figure S14 25
Appendix Figure S15 27
Appendix Figure S16 28
Appendix Figure S17 29
Appendix Figure S18 31
Appendix Figure S19 33
Appendix Figure S20 34
Appendix Figure S21 35
Appendix Figure S22 36
Appendix Figure S23 38
Appendix Figure S24 40

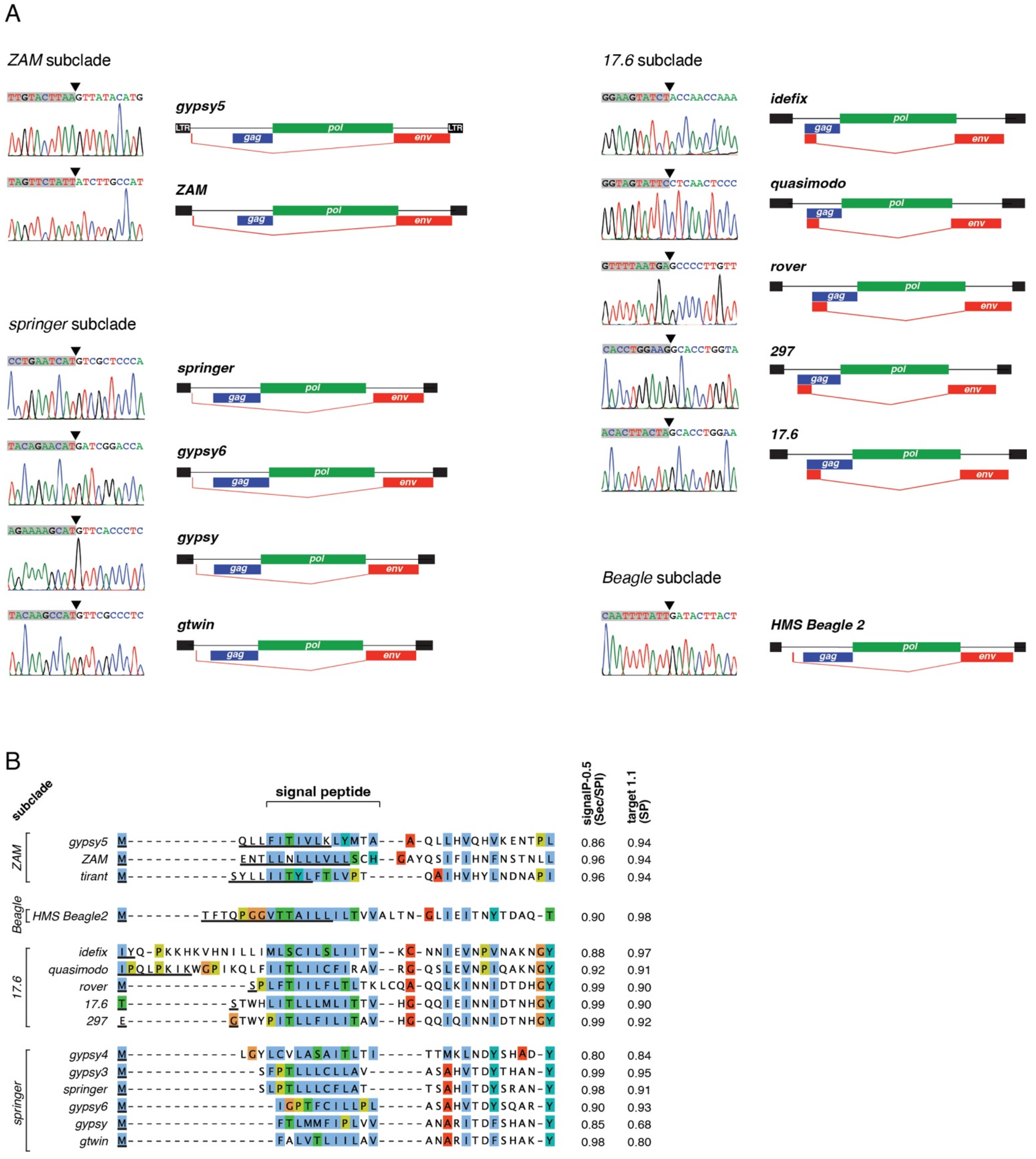

Appendix Figure S1. ***env-F*** **splice patterns among iERVs.**

A, Shown are Sanger sequencing chromatograms, centered on the *env-F* splice junction of the indicated iERVs, from subcloned PCR products obtained from first strand cDNA prepared from total RNA of ovaries lacking somatic piRNA control (*tj*-Gal4 driven *vreteno*^{GD} RNAi). Also shown are cartoons of the corresponding retrovirus showing the *env-F* splice pattern, drawn to scale. **B,** Protein sequence alignment of the spliced N-terminus of Env-F proteins from the indicated iERV consensus sequences. Underlined amino acids are encoded by the upstream exon, the signal peptide is indicated, the predicted cleavage site (red) and the respective scores using two different prediction algorithms are shown.

Functional retroviral *envelope-F* splice junctions and their conservation patterns

**ZAM subclade**	upstream	downstream	
ZAM (known, this study)	TAGTTCTATTGTAAGTAGTT	TGCATTCCAGATCTTGCCAT	seq confirmed
gypsy5 (this study)	TTGTACTTAAGTGAGTAGAG	ATCATTTCAGGTTATACATG	seq confirmed (distinct from consensus)
tirant (known)	TTACCTACCTGTAAGTAAAC	ACCATTTCAGGTTCACCCTT	not deregulated

**Idefix subclade**	upstream	downstream	
quasimodo (this study)	GGTAGTATTCGTAAGTTTGT	ACAATTCCAGCTCAACTCCC	seq confirmed
idefix (known/this study)	GGAAGTATCTGTAAGTTTAT	ATAATTCCAGACCAACCAAA	seq confirmed
297 (this study)	CACCTGGAAGGTAACCAATC	AATTTCACAGGCACCTGGTA	seq confirmed
rover (this study)	GTTTTAATGAGTAAGTTAGA	CATTTTACAGGCCCCTTGTT	seq confirmed
17.6 (this study)	ACACTTACTAGTAAGCTTGA	AATTTCACAGGCACCTGGCA	seq confirmed

**Springer subclade**	upstream	downstream	
active			
gtwin (this study)	TACAAGCCATGTAAGTTTGA	TGGTTTGCAGGTTCGCCCTC	seq confirmed
gypsy (known, this study)	AGAAAAGCATGTAAGTTTTA	TAATTTCCAGGTTCACCCTC	seq confirmed
gypsy6 (this study)	TACAGAACATGTACATCTTC	AATTTCACAGGATCGGACCA	seq confirmed
springer (this study)	CCTGAATCATGTAAGTGGGA	CACGTTACAGGTCGCTCCCA	seq confirmed
inactive			
gypsy2 (this study)	CAAAAAAAATGTAAGTGGGC	TCACCCACAGGACCAATCTT	predicted/not deregulated
gypsy3 (this study)	CACAGATTATGTAAGTGAGC	CATATTACAGGTCGTTTCCA	predicted/not deregulated
gypsy4 (this study)	TGAAATACATGTAAGTGATT	TTTATTACAGGTTAGGATAT	predicted/not deregulated
gypsy10 (this study)	AATATAACATGTAAGTGAGA	TCGCACTTAGGTTAGGAAAC	predicted/not deregulated

**Beagle subclade**	upstream	downstream	
HMS Beagle2 (this study)	CAATTTTATTGTGAGACAAG	TTACTTTCAGGATACTTACT	seq confirmed

Appendix Figure S2. Conservation of *env-F* splice junctions.
Shown are sequence alignments of the experimentally identified *env-F* splice donor and acceptor junctions and their local conservation within the different iERV subclades (intronic nucleotides in red are conserved in at least 80% of all sequences).

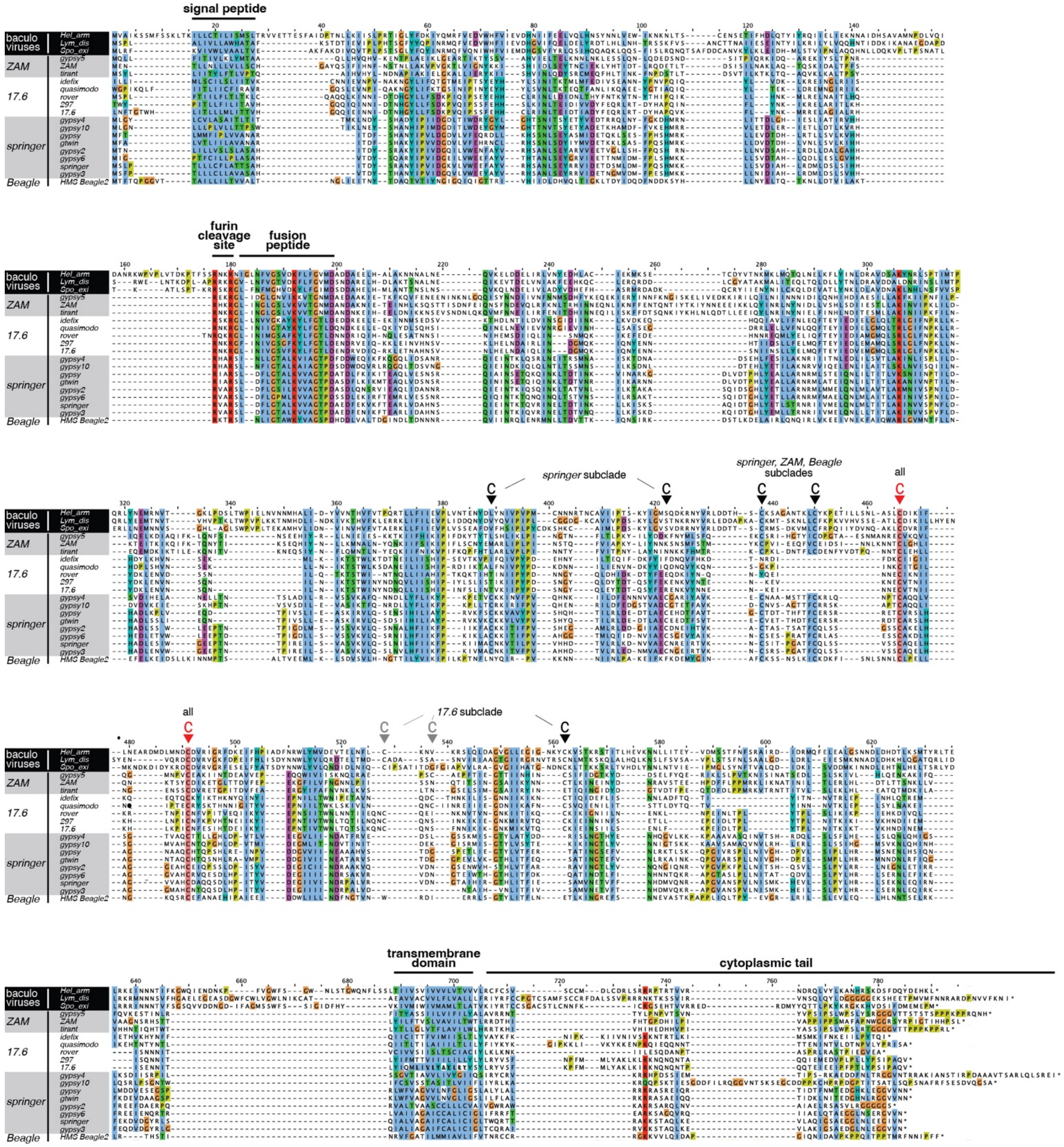

Appendix Figure S3. Similarity between iERV Env-F proteins and baculovirus F-type fusion glycoproteins.

Shown is a protein sequence alignment of all *Drosophila melanogaster* iERV spliced Envelope-F proteins together with three F-type fusion glycoproteins from baculoviruses isolated from indicated lepidopteran species. N-terminal signal peptides, furin cleavage sites, fusion peptides, conserved cysteine residues presumably involved in di-sulfide bond formation, transmembrane domains, and C-terminal cytoplasmic tails are indicated.

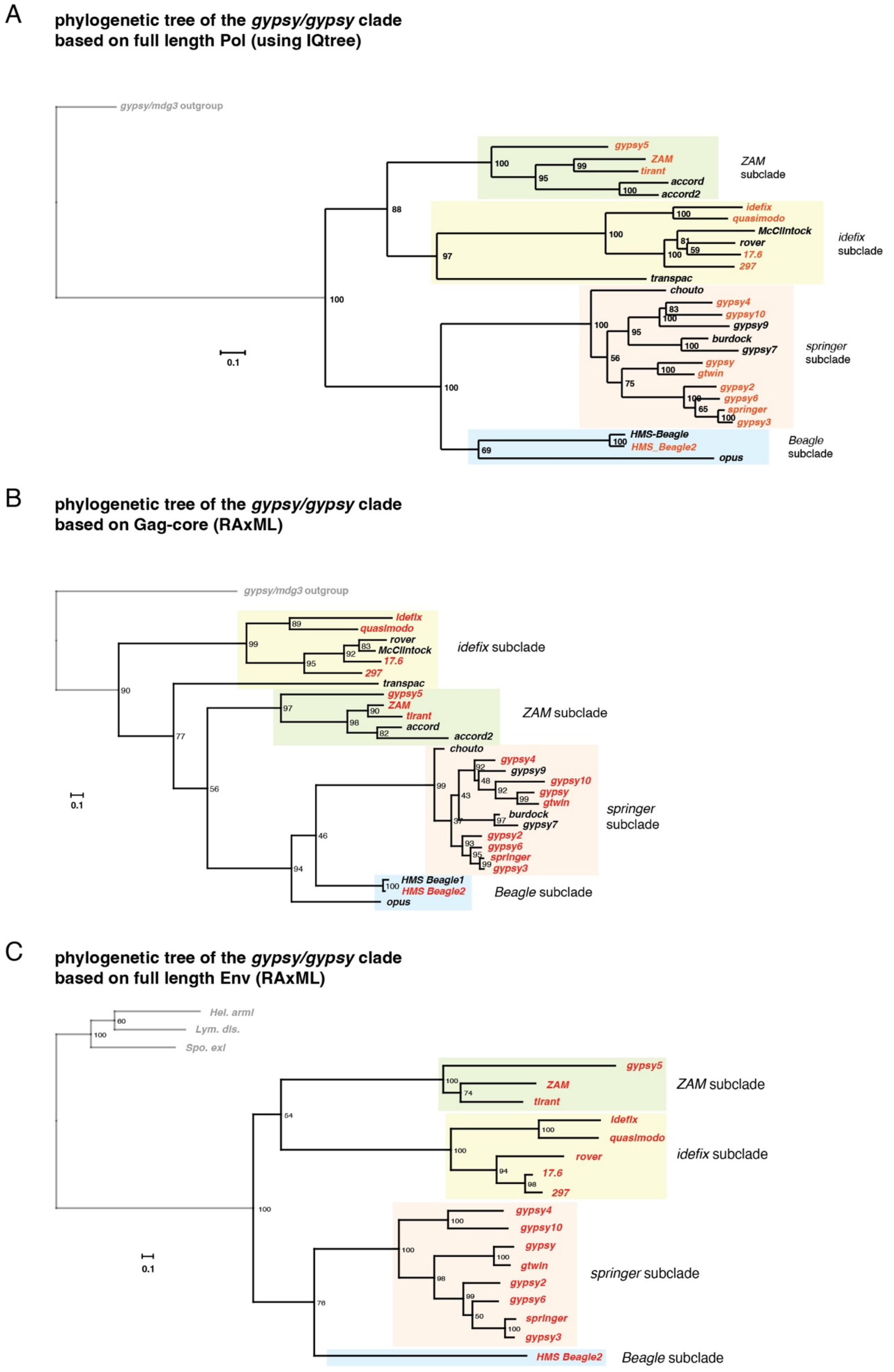

Appendix Figure S4 . Phylogenetic trees for iERV Gag, Pol, and Env proteins.

A, Shown are IQ-TREE estimated phylogenetic trees (scale indicates amino acid substitutions per site) from the full-length iERV Pol alignment with the *gypsy/mdg3* outgroup as in Fig. 1a. Node labels reflect 1000 ultrafast bootstraps. Lineages with full length *env-F* are shown in red, those with mutated/lost *env-F* in black; subclades are color-shaded. **B, C,** Shown are RAxML-estimated phylogenetic trees based on Gag core domain and full-length Env protein alignments from all iERV lineages (respective outgroups in grey; labelling as in (A)). Note the overall congruence of the tree architectures in (A-C), despite the fact that the Gag core domain has diverged more than Pol and Env during evolution (see Appendix Figure S16A).

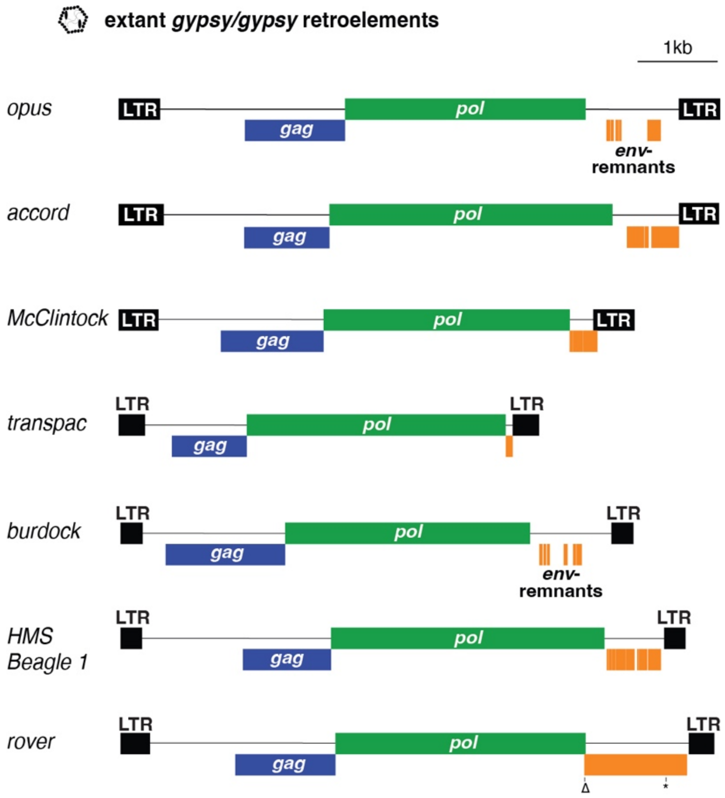

Appendix Figure S5. Retroelement revertants among iERVs harbor *env-F* fragments.
Cartoons (drawn to scale) of active iERV retroelements with identified fragments corresponding to *env-F* coding sequence fragments indicated in orange (*env-F* remnants). Details of the *env-F* fragments are shown in Appendix Figure S6. *rover*: The triangle indicates the 73bp deletion in some structurally intact retro-element insertions, the * indicates a single A nucleotide insertion leading to a frameshift in the *env-F* ORF in retro-element insertions.

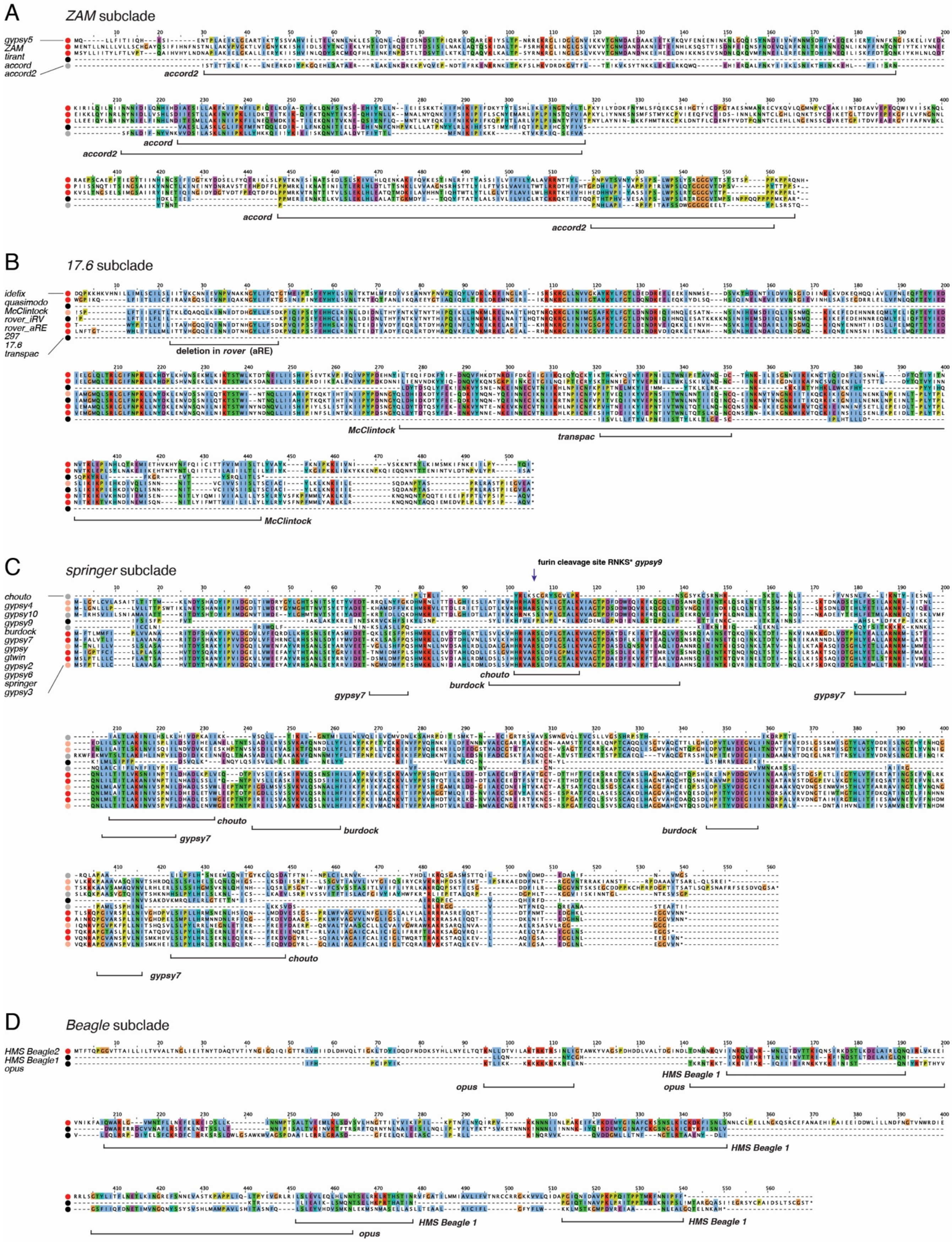

Appendix Figure S6. Sequence remnants of *env-F* in iERV retroelement revertants.

MACSE protein sequence alignments of Env-F and Env-F remnants within each iERV subclade. Dots on the left classify species as active retroviruses (dark red), inactive retroviruses (light red), active retroelements (black), or inactive retroelements (grey). Bracketed regions indicate *env-F* remnants present in derived LTR retroelements lacking full-length *env-F* genes. **A-D,** Shown are Env-F alignments for the *ZAM-* (A), *idefix-* (B*)*, *springer* (C) and *Beagle*-subclade (D). In (B) the Env-F sequences of two *rover* variants are shown: *rover* inactive retrovirus (*rover*-iRV) and *rover* active retroelement (aRE).

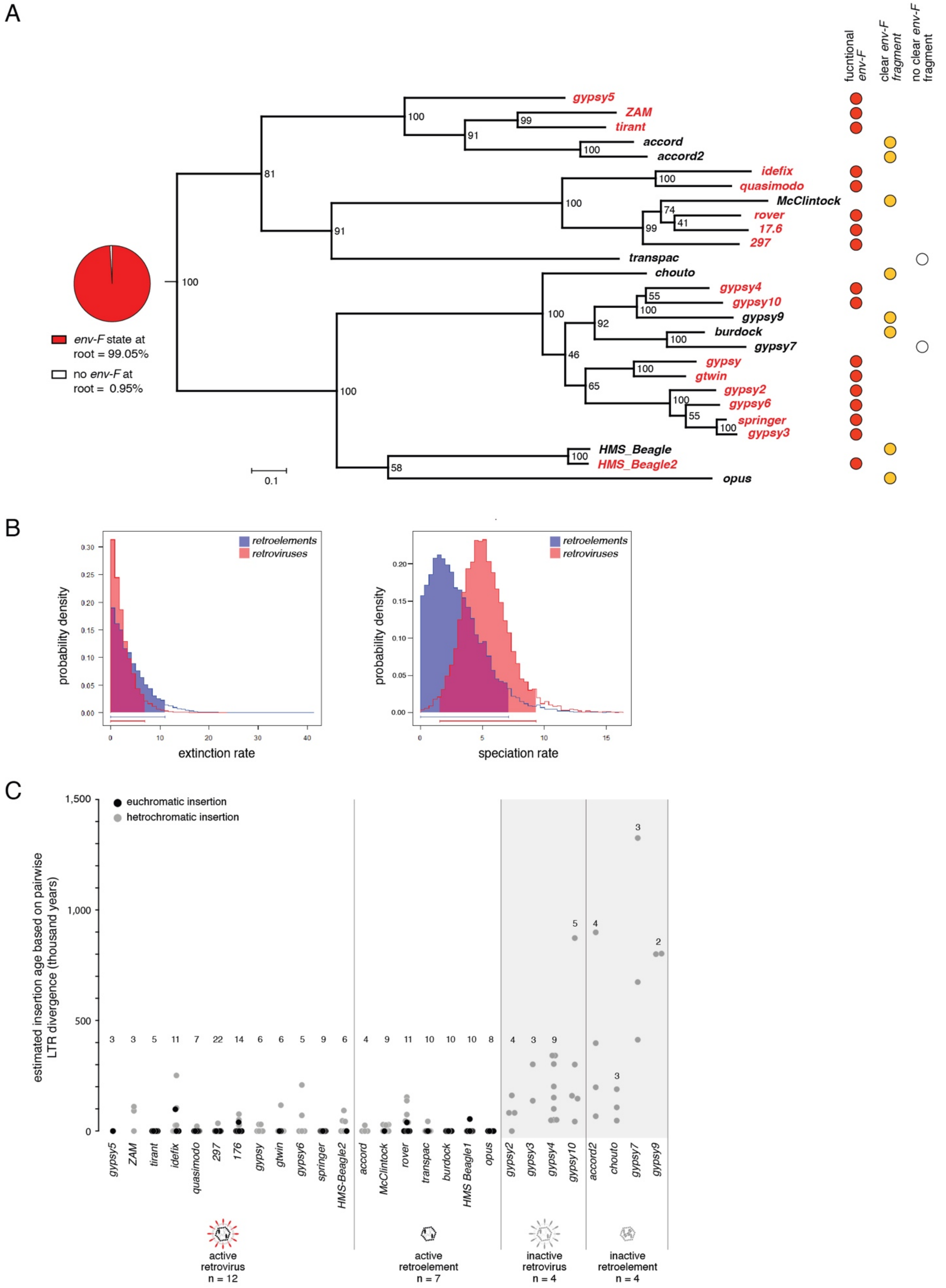

Appendix Figure S7. Ancestral character, diversification, and estimated age of iERV insertions in the *D. melanogaster* genome.

A, Shown is the Pol sequence alignment-based phylogenetic tree (RAxML; scale indicates amino acid substitutions per site) without outgroup and terminal branch-associated *env-F* character states. Each lineage/terminal branch was assigned the character state "*envelope* present", which included functional *envelope* (red circles) or non-functional *envelope* fragment (orange circles) or the character state "envelope absent" (open circles for *transpac* and *gypsy7*). Ancestral character reconstruction using Diversitree (BiSSE) estimates an *env-F*-containing state at the root of the iERV clade with 99.1% probability. **B,** Shown are BiSSE-based Bayesian estimated differences in extinction (left panel) or speciation rates (right panel; each lineage/terminal branch was assigned with the character state 'retrovirus' (functional *envelope*) or 'retroelement' (non-functional *envelope*)). Estimates were based on the same tree as shown in (A). The estimated speciation rates are higher for retroviruses, but not statistically significant. The estimated extinction rates are not different. **C,** Shown are estimated insertion ages (in thousands of years ago; TYA) calculated based on pairwise LTR divergence of all analyzed iERV insertions, grouped into active and inactive retroviruses, and active and inactive retroelements (numbers above indicate the number of analyzed insertions).

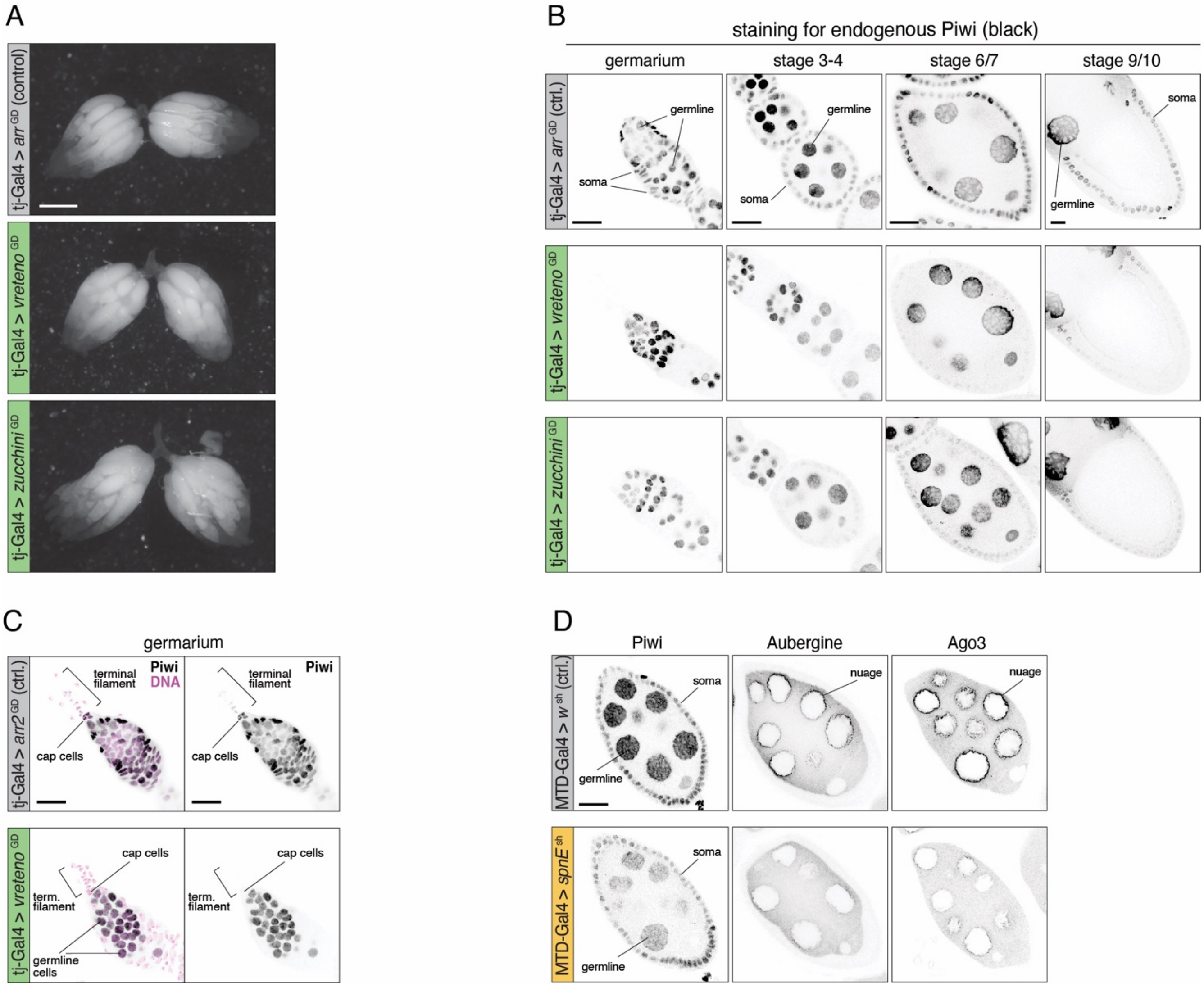

Appendix Figure S8. Validation of piRNA pathway knockdowns in ovaries.

A, Ovarian morphology is not altered upon piRNA pathway knockdowns in the soma (*tj*-Gal4 driven knockdown against *vreteno* or *zucchini* compared to control knockdown against *arrestin2*). Scale bar: 500 µm. **B,** Antibody staining against Piwi (black) in ovaries (developmental stages indicated) expressing long dsRNA hairpin constructs (VDRC lines) against *vreteno* or *zucchini* under *tj*-Gal4 control compared to control ovaries (Piwi protein levels are specifically reduced in somatic cells; scale bars: 20µm). **C,** As in (B) but showing terminal filament cells at the anterior tip of the germarium, which express Piwi at low but detectable levels in wild-type ovaries (scale bars: 20µm). **D,** Antibody stainings against Piwi, Aub, and Ago3 (black) are shown in control ovaries and in ovaries expressing a short hairpin RNA (TRIP lines) against *spn-E* specifically in germline cells under control of the MTD-Gal4 driver (scale bar: 20µm; *aub* and *aub*+*ago3* knockdown conditions are described in (Senti *et al*, 2015)). Loss of nuage localization for Aub/Ago3 and reduced levels for Piwi indicate a strong disruption of the germline piRNA pathway.

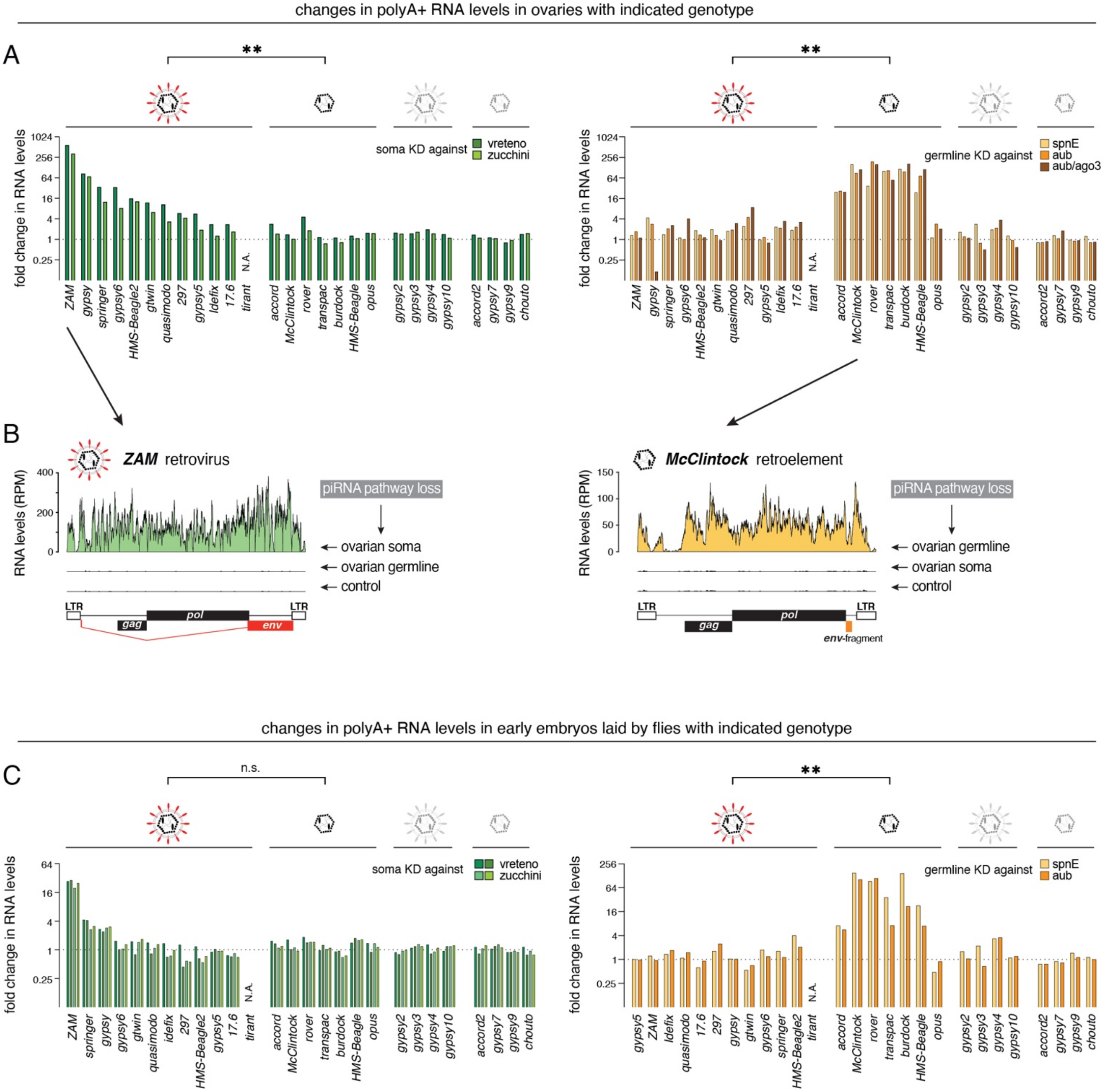

Appendix Figure S9. Changes in iERV transcript levels in ovaries or early embryos upon loss of the piRNA pathway.

A, Fold changes of iERV poly-A$^+$ RNA levels are shown in ovaries lacking the piRNA pathway in the soma (left) or in the germline (right) compared to control ovaries. **B,** Normalized transcript levels (RPM) of the representative retrovirus *ZAM* and the retroelement *McClintock* are shown in control ovaries or ovaries lacking somatic or germline piRNA pathway control (genotypes: *tj*-Gal4 > *vreteno*^{GD}, *MTD*-Gal4 > sh$^{aub+ago3}$, *tj*-Gal4 > *arrestin2*^{GD} for *ZAM*; and *MTD*-Gal4 > sh$^{aub+ago3}$, *tj*-Gal4 > *vreteno*^{GD}, *MTD*-Gal4 > sh^{white} for *McClintock*). **C,** Fold changes of iERV poly-A$^+$ RNA levels are shown in early embryos laid by flies lacking piRNA pathway control in the soma (left) or germline (right). The *tirant* retrovirus cannot be analyzed as it is not present in some of our experimental strains (N.A.). iERVs are grouped into active retroviruses, active retroelements, inactive retroviruses and inactive retroelements. Statistical significance (** indicates p < 0.01) was calculated according to Mann-Whitney (two-tailed test, performed separately for each genotype.)

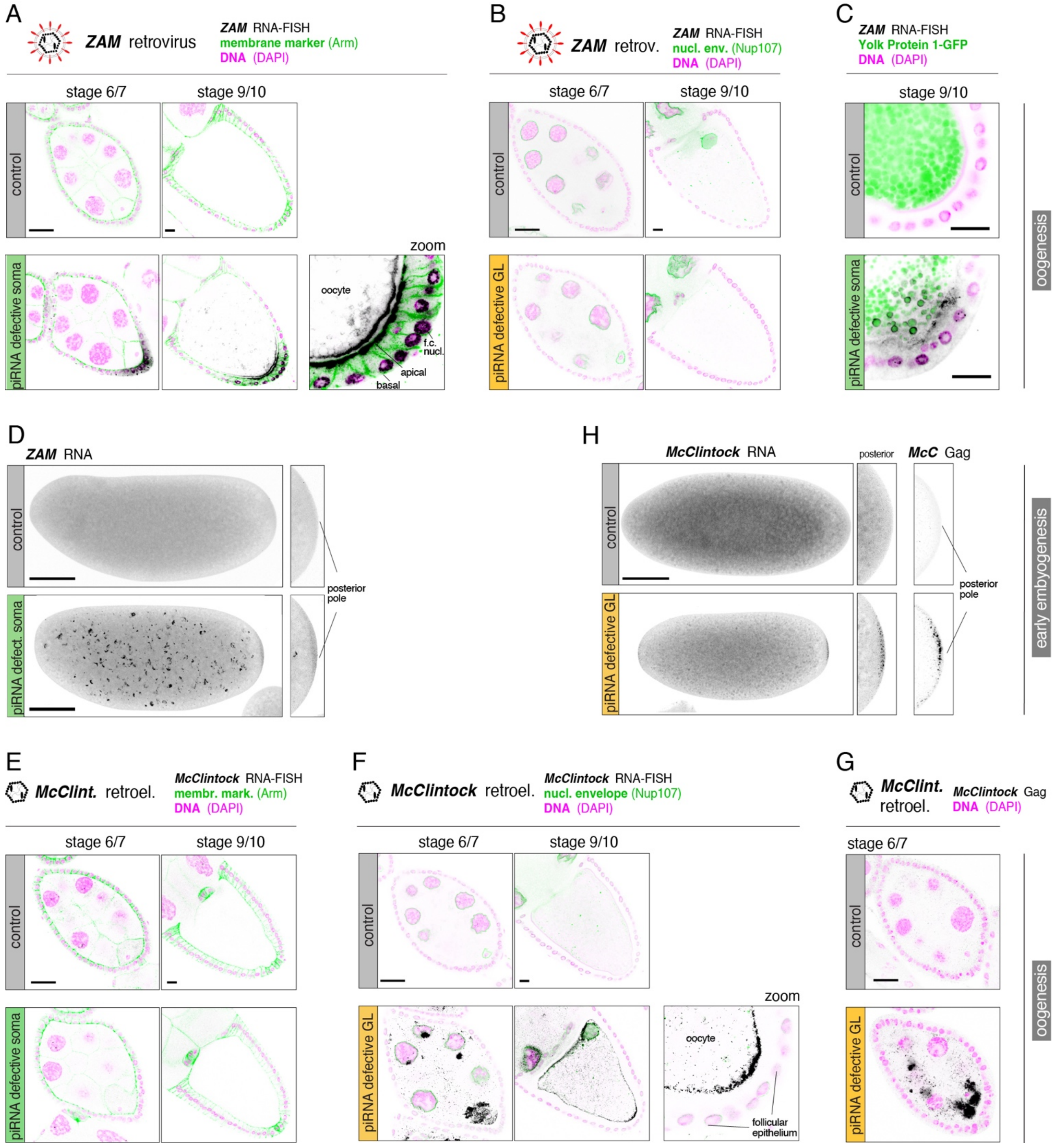

Appendix Figure S10. Replication strategies of infectious versus non-infectious iERVs.

A, RNA-smFISH based expression analysis for the retrovirus *ZAM* in egg chambers of indicated stage from control ovaries or from ovaries with defective somatic piRNA pathway (*tj*-Gal4 driven dsRNA hairpin against *vreteno* or against *arrestin2* for control; scale bars: 20µm; cell outlines visualized with an anti-Armadillo antibody and DNA with DAPI). The enlarged panel shows the posterior pole of the growing stage 9/10 oocyte with adjacent follicle cells (f.c.). **B,** Complete repression of the retrovirus *ZAM*, assessed by RNA smFISH, in control ovaries as well as in ovaries with defective germline piRNA pathway (genotypes are *MTD*-Gal4 driven shRNAs against *aub*+*ago3* or *white*; DAPI labels nuclei (magenta), *ZAM* RNA is in black, GFP-Nup107 labels the nuclear envelope in green; scale bars: 20µm). **C,** Same as (A), but yolk granules labelled with YP1-GFP in green. Scale bar: 20 µm. **D,** RNA-smFISH based expression analysis for *ZAM* in pre-blastoderm embryos with less than 32 nuclei laid by females

lacking somatic piRNA pathway control (genotypes: *tj*-Gal4 > *vreteno*^{GD} or *arrestin2*^{GD}*;* images show maximum intensity Z-projections; scale bars: 100µm). Enlarged panels show accumulation of FISH signal at the posterior pole. **E,** Lack of expression of the *McClintock* retroelement, assessed by RNA smFISH, in control ovaries as well as in ovaries with a defective somatic piRNA pathway (genotypes are *tj*-Gal4 driven dsRNA hairpins against *vreteno* or *arrestin2* as control; DAPI labels nuclei (magenta), *McClintock* RNA is shown in black, anti-Armadillo labels cell outlines in green; scale bars: 20µm). **F,** Same as (A) but with RNA-smFISH against the *McClintock* retroelement in ovaries with defective germline piRNA pathway (*MTD*-Gal4 driven shRNA against *aub*+*ago3* or against *white* for control). Scale bar: 20 µm. **G,** *McClintock* Gag in stage 6/7 egg chamber detected by immunofluorescence in ovaries with defective germline piRNA pathway (bottom) or control ovaries (top). Note the accumulation of *McClintock* capsid protein in the developing and transcriptionally inactive oocyte (scale bar: 20µm). **H,** RNA-smFISH based expression analysis for *McClintock* in pre-blastoderm embryos with less than 32 nuclei laid by females lacking germline piRNA pathway control (genotypes: *MTD*-Gal4 driven shRNA against *aub* or *white;* images show maximum intensity Z-projections; scale bars: 100µm). Enlarged panels show accumulation of FISH signal at the posterior pole (also shown with an antibody staining against *McClintock* Gag). We note that some panels in Appendix Figure S10 were intentionally reused from Figure 2 to allow the direct side-by-side comparison of control and genetic depletion of the piRNA pathway.

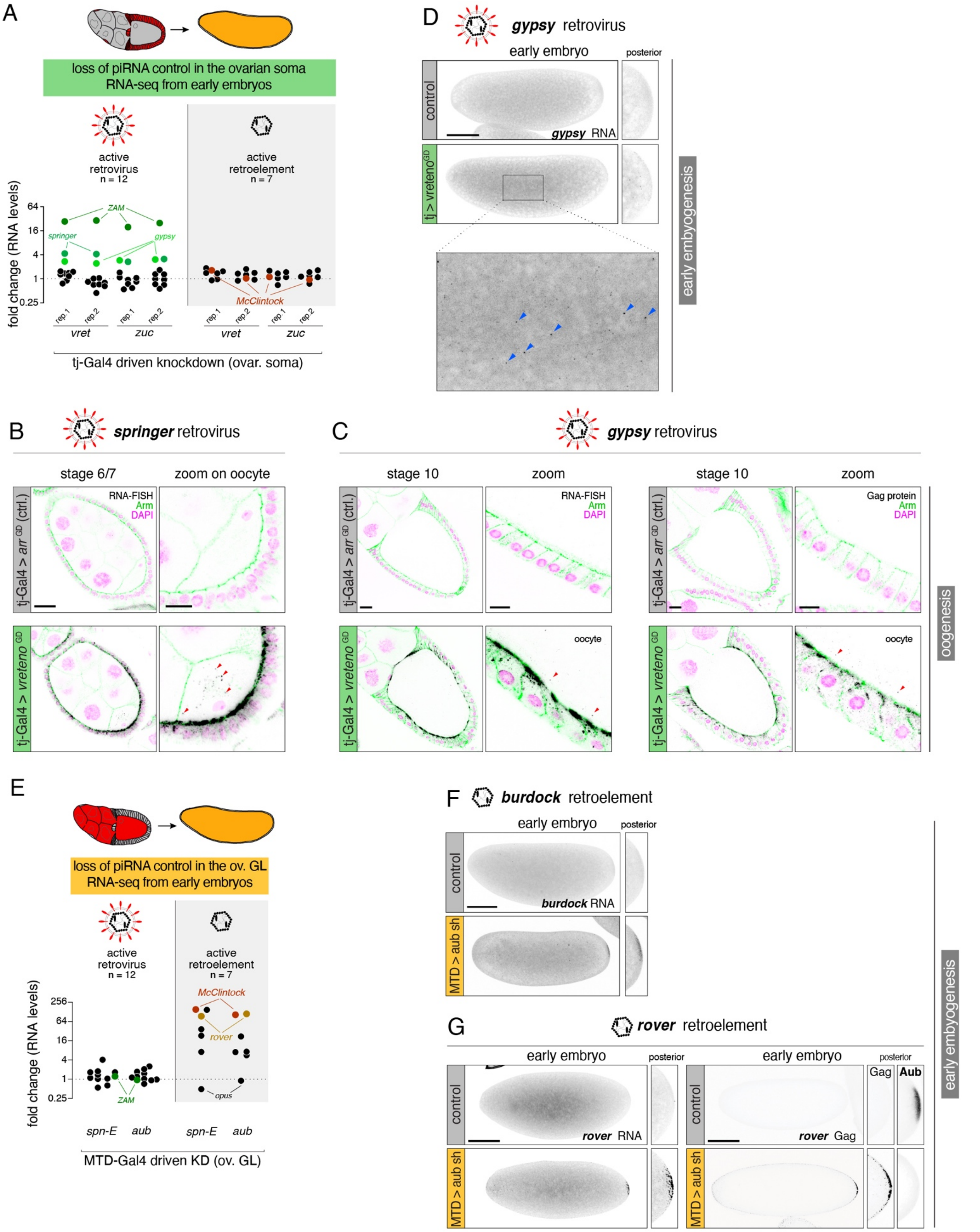

Appendix Figure S11. De-repression of iERVs in ovaries or early embryos upon loss of the piRNA pathway. **A,** Jitter plot showing fold changes in iERV steady-state polyA^{+} RNA levels in 0-60 min old, dechorionated embryos laid by mothers with defective somatic piRNA pathway versus embryos laid by control mothers (RNAi

and GAL4 strains used for transgenic RNAi are indicated; replicates are biological replicates). **B, C** Detection of *springer* (B) or *gypsy* (C) transcripts by RNA smFISH or *gypsy* Gag protein in egg chambers of indicated age and genotype. DAPI labels nuclei (magenta), anti-Armadillo staining labels cell outlines in green, red arrowheads point to RNA smFISH signal (black) in the developing oocyte, indicating soma-to-germline transfer (scale bars: 20µm for full egg chambers and 10µm for the enlarged region). **D,** Detection of *gypsy* transcripts by RNA-smFISH in pre-blastoderm embryos (<32 nuclei), laid by mothers with defective somatic piRNA pathway (maternal genotype: *tj*-Gal4 > *vreteno*^{GD} or *arrestin2*^{GD}*;* scale bar: 100µm). Images are maximum intensity Z-projections of confocal stacks. Individual smFISH dots are shown in the magnified image below. **E,** Jitter plot showing fold changes in iERV steady-state polyA^{+} RNA levels in 0-60 min old, dechorionated embryos laid by mothers with defective germline piRNA pathway compared to those laid by control mothers (RNAi and GAL4 strains used for transgenic RNAi are indicated; replicates are biological replicates). **F, G,** Detection of *burdock* (F) or *rover* (G) transcripts by RNA-smFISH in pre-blastoderm embryos (<32 nuclei) laid by mothers with defective germline piRNA pathway (maternal genotype: *MTD*-Gal4 driven shRNA against *aub* or *white;* scale bars: 100µm). In the case of *rover*, the same analysis was also performed using an anti-*rover* Gag antibody and with an anti-Aub staining serving as a knockdown control (G). Images are maximum intensity Z-projections of confocal stacks. The enlarged images show the posterior pole where the primordial germ cells will form.

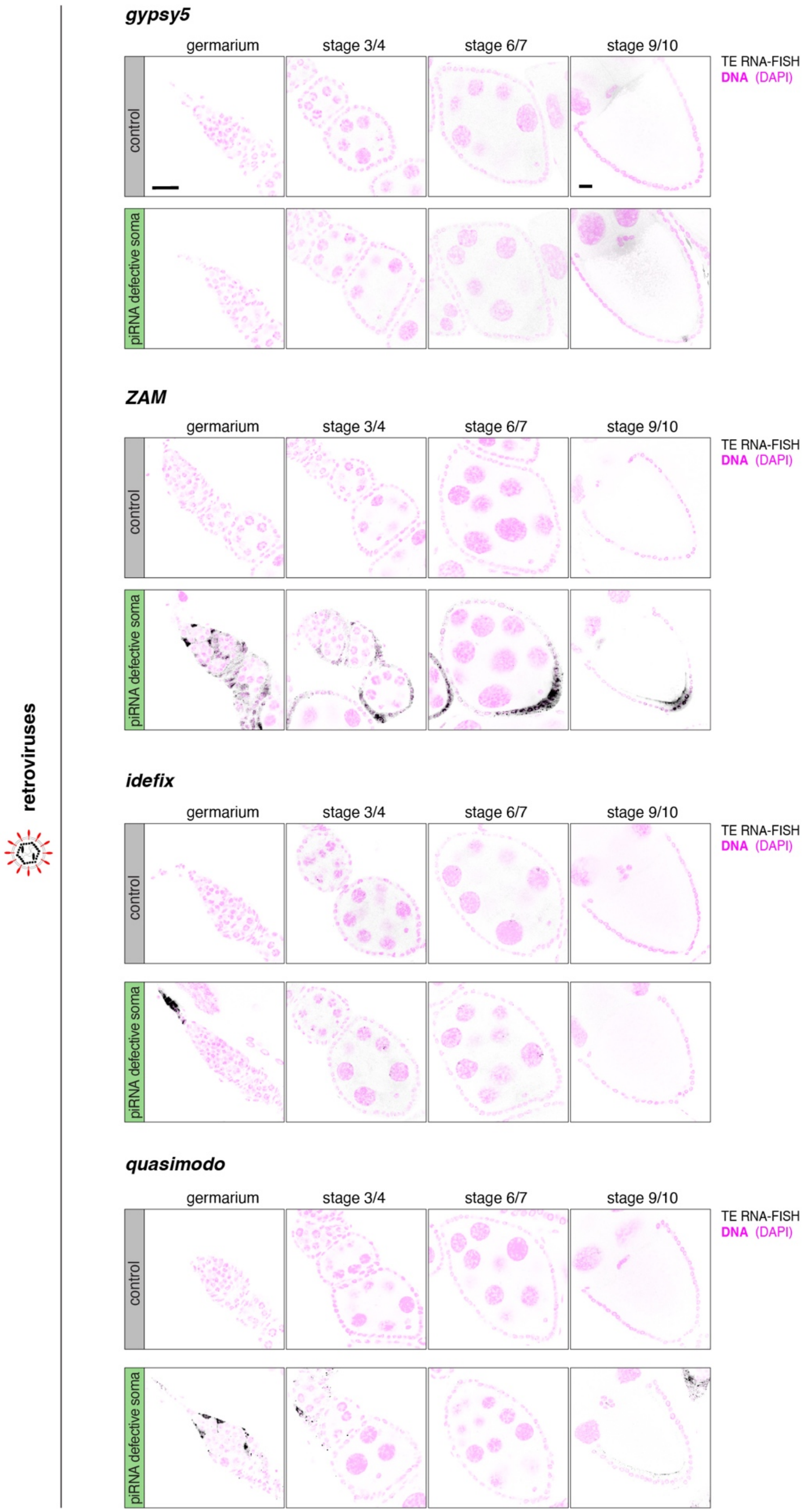
retroviruses
gypsy5
ZAM
idefix
quasimodo
germarium
stage 3/4
stage 6/7
stage 9/10
control
piRNA defective soma
TE RNA-FISH
DNA (DAPI)

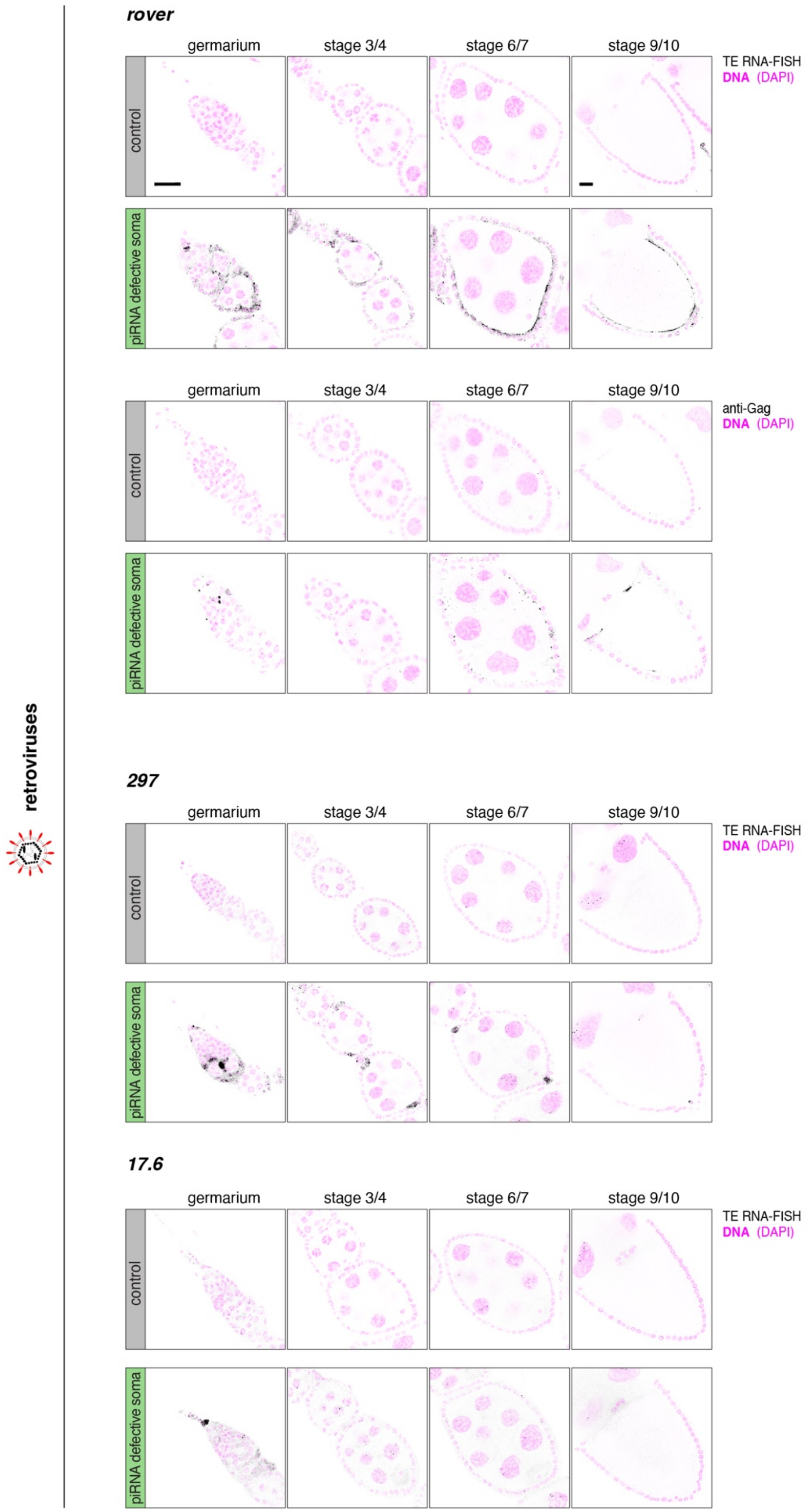
retroviruses
rover
germarium
stage 3/4
stage 6/7
stage 9/10
TE RNA-FISH
DNA (DAPI)
control
piRNA defective soma
anti-Gag
DNA (DAPI)
297
17.6

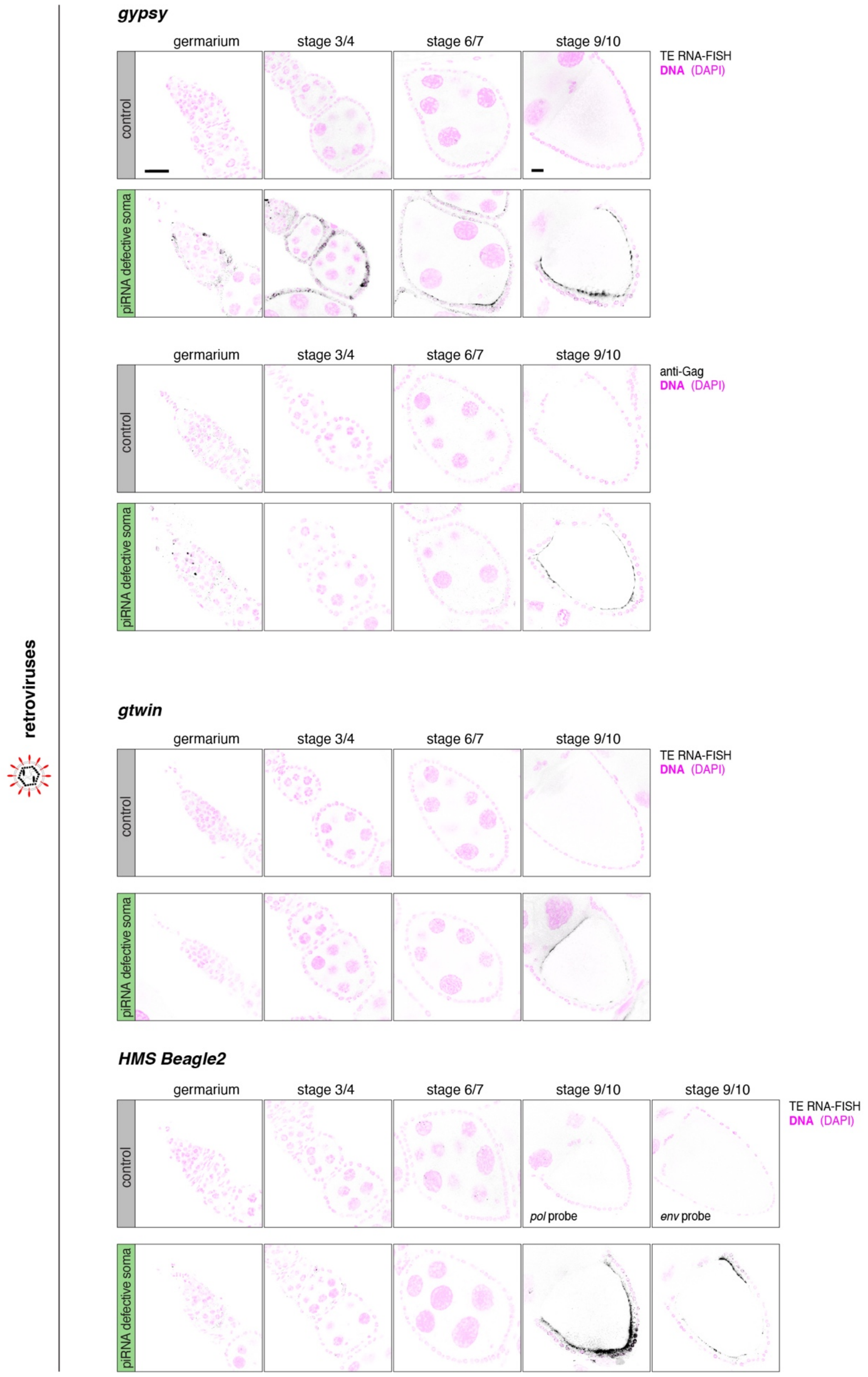
retroviruses
gypsy
germarium
stage 3/4
stage 6/7
stage 9/10
TE RNA-FISH
DNA (DAPI)
control
piRNA defective soma
germarium
stage 3/4
stage 6/7
stage 9/10
anti-Gag
DNA (DAPI)
control
piRNA defective soma
gtwin
germarium
stage 3/4
stage 6/7
stage 9/10
TE RNA-FISH
DNA (DAPI)
control
piRNA defective soma
HMS Beagle2
germarium
stage 3/4
stage 6/7
stage 9/10
stage 9/10
TE RNA-FISH
DNA (DAPI)
control
pol probe
env probe
piRNA defective soma

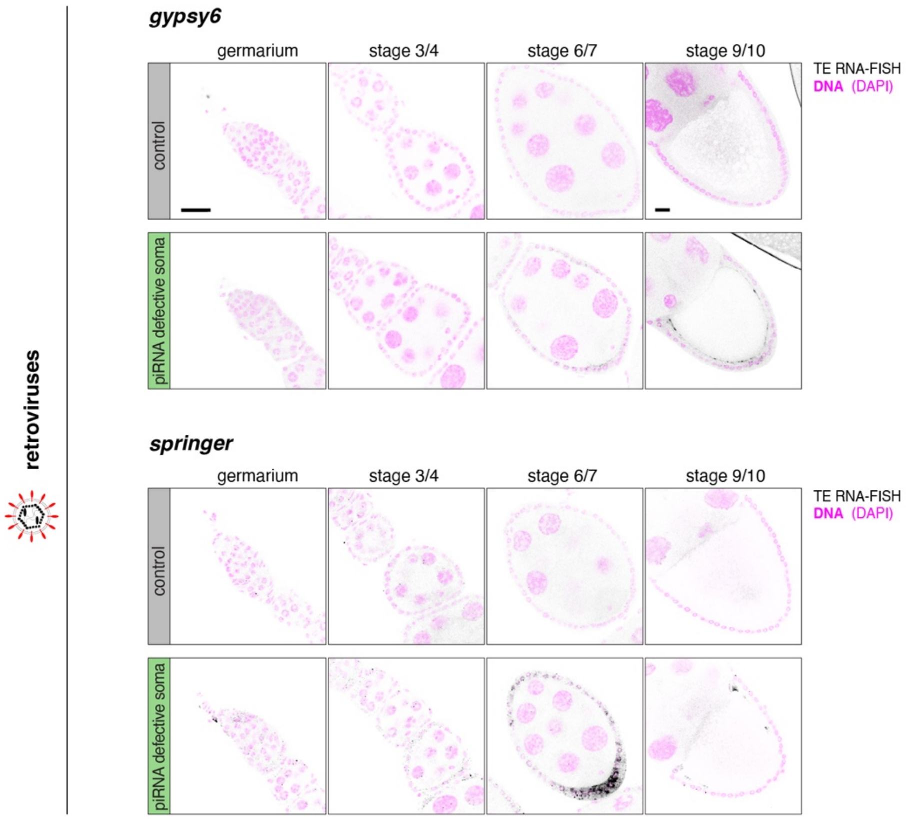
retroviruses
gypsy6
germarium
stage 3/4
stage 6/7
stage 9/10
TE RNA-FISH
DNA (DAPI)
control
piRNA defective soma
springer
germarium
stage 3/4
stage 6/7
stage 9/10
TE RNA-FISH
DNA (DAPI)
control
piRNA defective soma

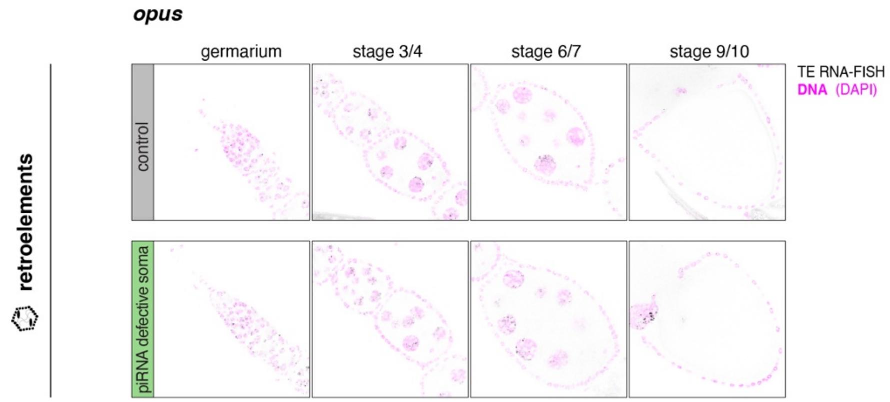
retroelements
opus
germarium
stage 3/4
stage 6/7
stage 9/10
TE RNA-FISH
DNA (DAPI)
control
piRNA defective soma

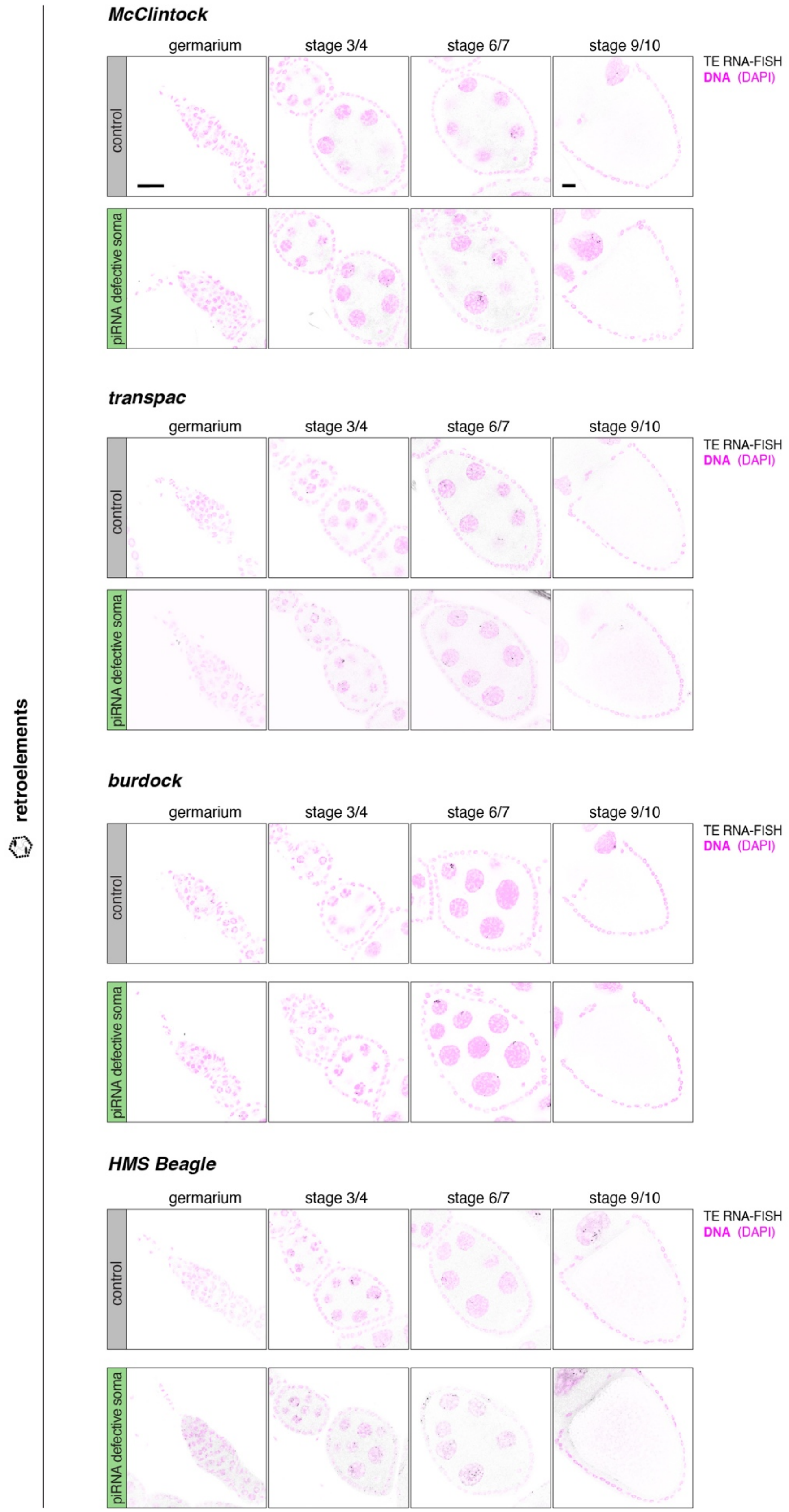
retroelements
McClintock
germarium
stage 3/4
stage 6/7
stage 9/10
TE RNA-FISH
DNA (DAPI)
control
piRNA defective soma
transpac
germarium
stage 3/4
stage 6/7
stage 9/10
TE RNA-FISH
DNA (DAPI)
control
piRNA defective soma
burdock
germarium
stage 3/4
stage 6/7
stage 9/10
TE RNA-FISH
DNA (DAPI)
control
piRNA defective soma
HMS Beagle
germarium
stage 3/4
stage 6/7
stage 9/10
TE RNA-FISH
DNA (DAPI)
control
piRNA defective soma

Appendix Figure S12. Systematic expression analysis of all active iERV lineages in ovaries lacking somatic piRNA pathway control.

The complete set of smFISH experiments and immuno-fluorescence experiments against indicated iERVs (retroviruses and retroelements) is shown in control ovaries and in ovaries with defective piRNA pathway in the soma (all oogenesis stages are shown, scale bars: 20µm).

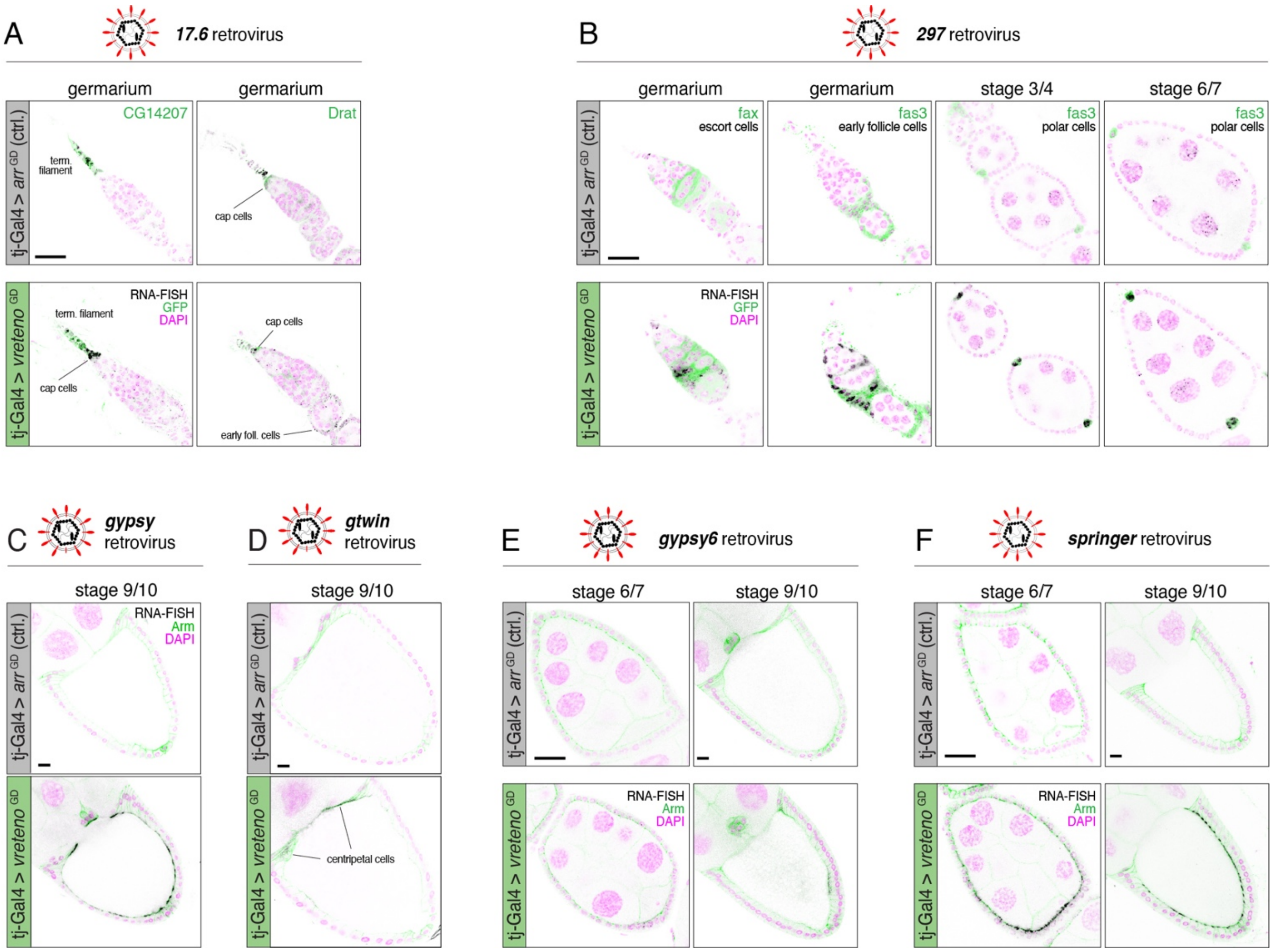

Appendix Figure S13. Niche expression of infectious iERVs in the ovarian soma.
A-F, Detection of indicated iERV transcripts by RNA-smFISH (black) in egg chambers of indicated oogenesis stages and genotypes. DAPI is shown in magenta, indicated GFP-traps (A, B) or anti-Armadillo staining (C-F) in green; scale bars: 20µm.

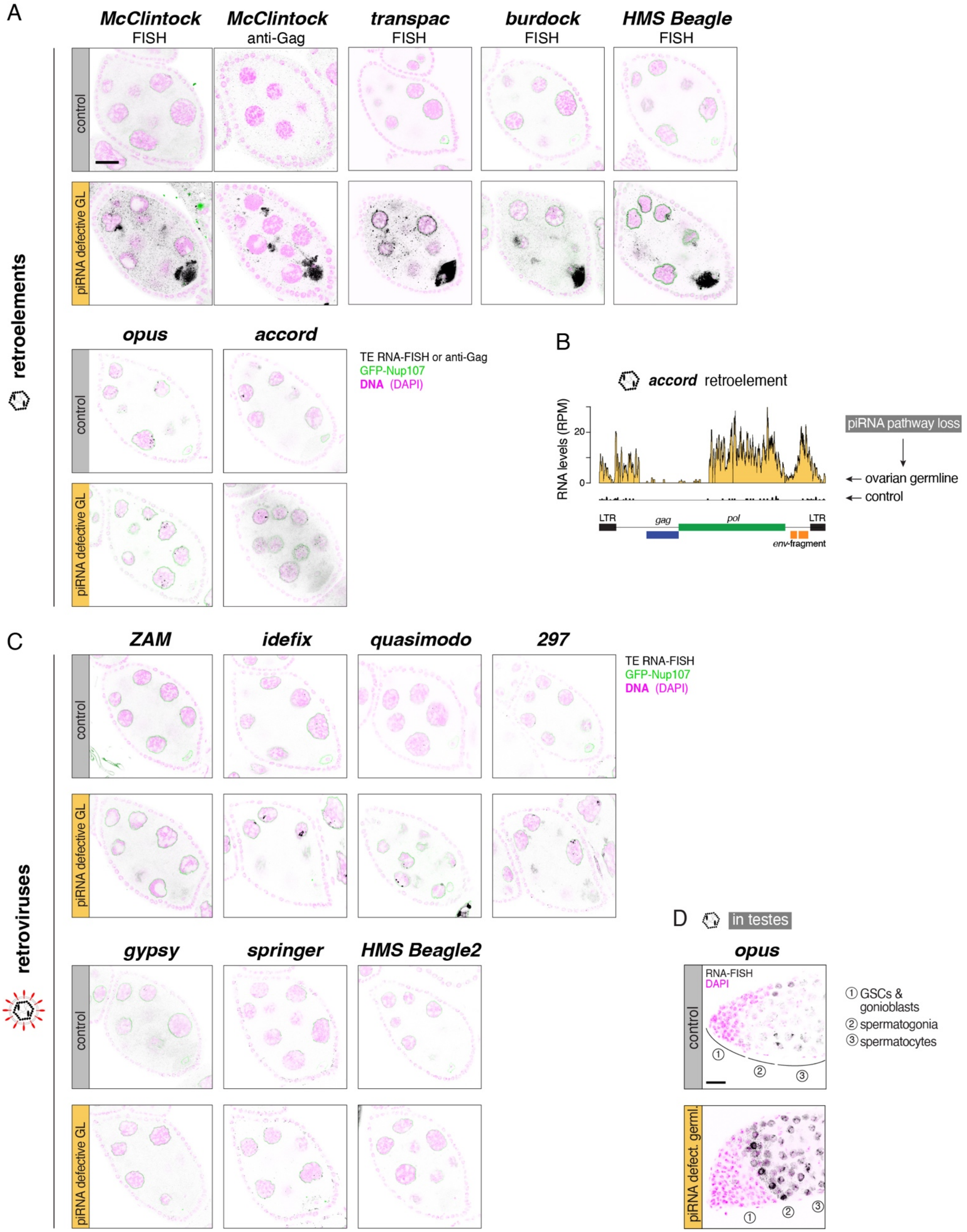

Appendix Figure S14. Systematic expression analysis of all active iERV lineages in ovaries lacking germline piRNA pathway control.

A, smFISH experiments and immuno-fluorescence experiments against indicated iERVs (retroelements) are shown in control ovaries and in ovaries with defective piRNA pathway in the germline (scale bars: 20µm). Only stage 6/7 egg chambers are shown, as other stages did not provide additional information. **B,** The increase in *accord* RNA levels assessed by poly-A^+ RNA-seq is shown in ovaries lacking piRNA pathway control in the germline. The gap in read coverage suggests that our experimental flies lack full-length *accord* insertions. **C,** Similar to (A) but for infectious retroviruses. **D,** RNA-smFISH based expression analysis of *opus* (black) in the anterior tip of testes lacking germline piRNAs compared to controls (*nanos*+*bam*-Gal4 driven shRNAs against *aub*+*ago3* or *white* as control; DAPI is shown in magenta; major germline cell types are indicated; scale bar: 20µm).

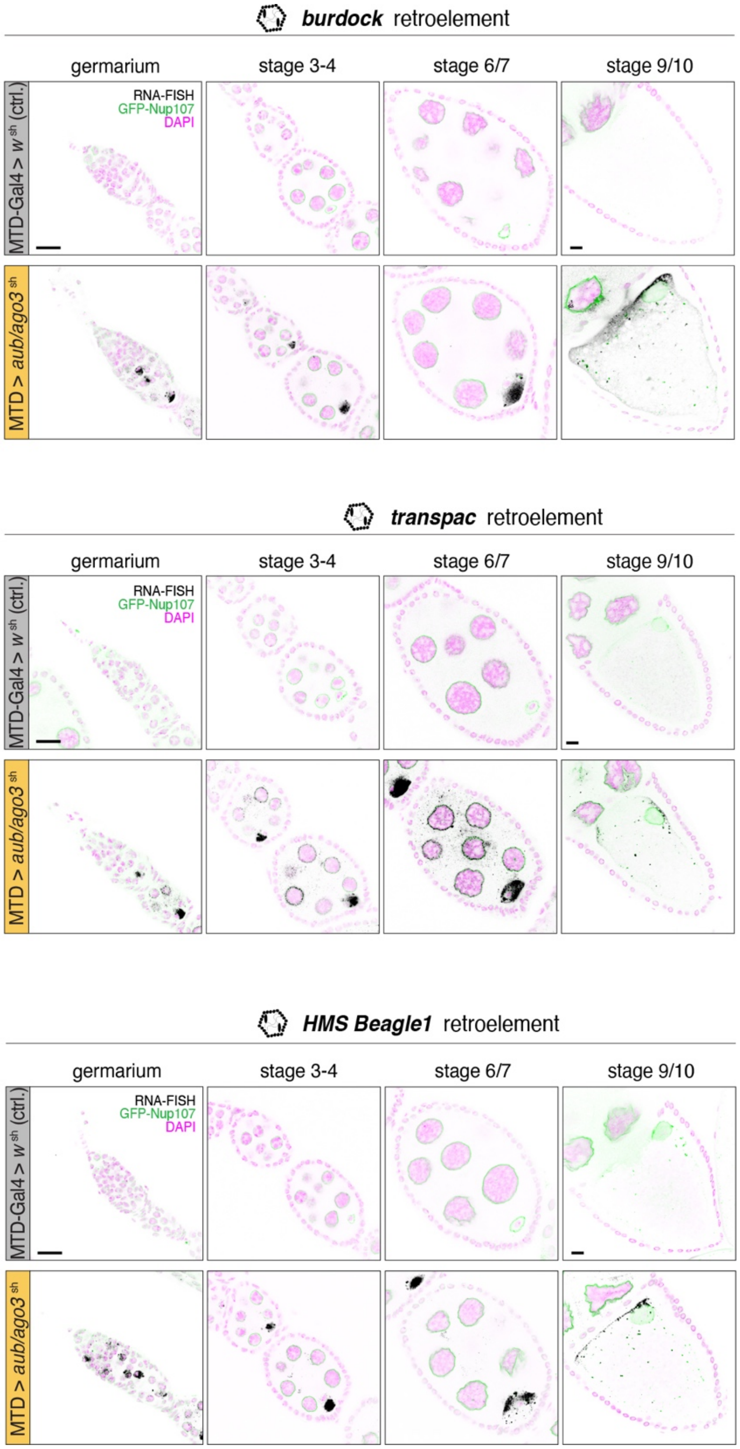

Appendix Figure S15. Germline specific expression of non-infectious iERVs in the ovarian germline. Detection of the indicated iERV transcripts by RNA-smFISH (black) in germaria or egg chambers of the indicated oogenesis stages and genotypes (DAPI labels nuclei (magenta), GFP-Nup107 labels nuclear envelopes (green); scale bars: 20µm). Retroelement transcripts start to be detectable in differentiating germline cystoblasts within the germarium and are enriched in the developing oocyte where they accumulate around the oocyte nucleus.

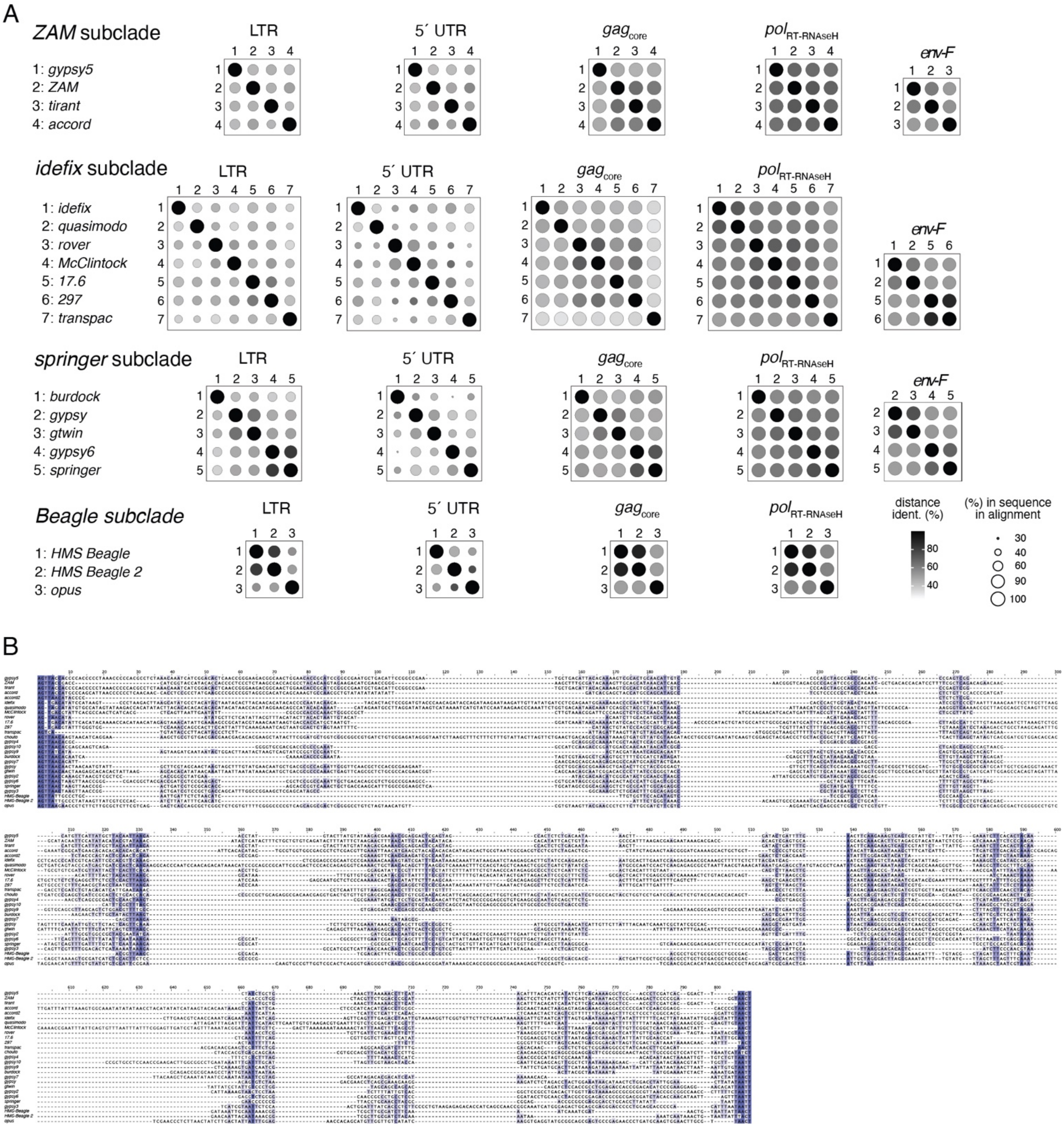

Appendix Figure S16. LTR and 5′ UTR sequence divergence among iERVs.

A, Pairwise identities between the DNA sequences for LTR, 5′ UTR, the protein domains Gag_{core}, $Pol_{RT\text{-}RNAseH}$, and full-length spliced Env-F are shown (circle size indicates percentage of nucleotides in aligned positions, grey scale indicates pairwise nucleotide identities among iERVs, separately for the indicated subclades). **B,** DNA sequence alignment of all iERV LTR sequences.

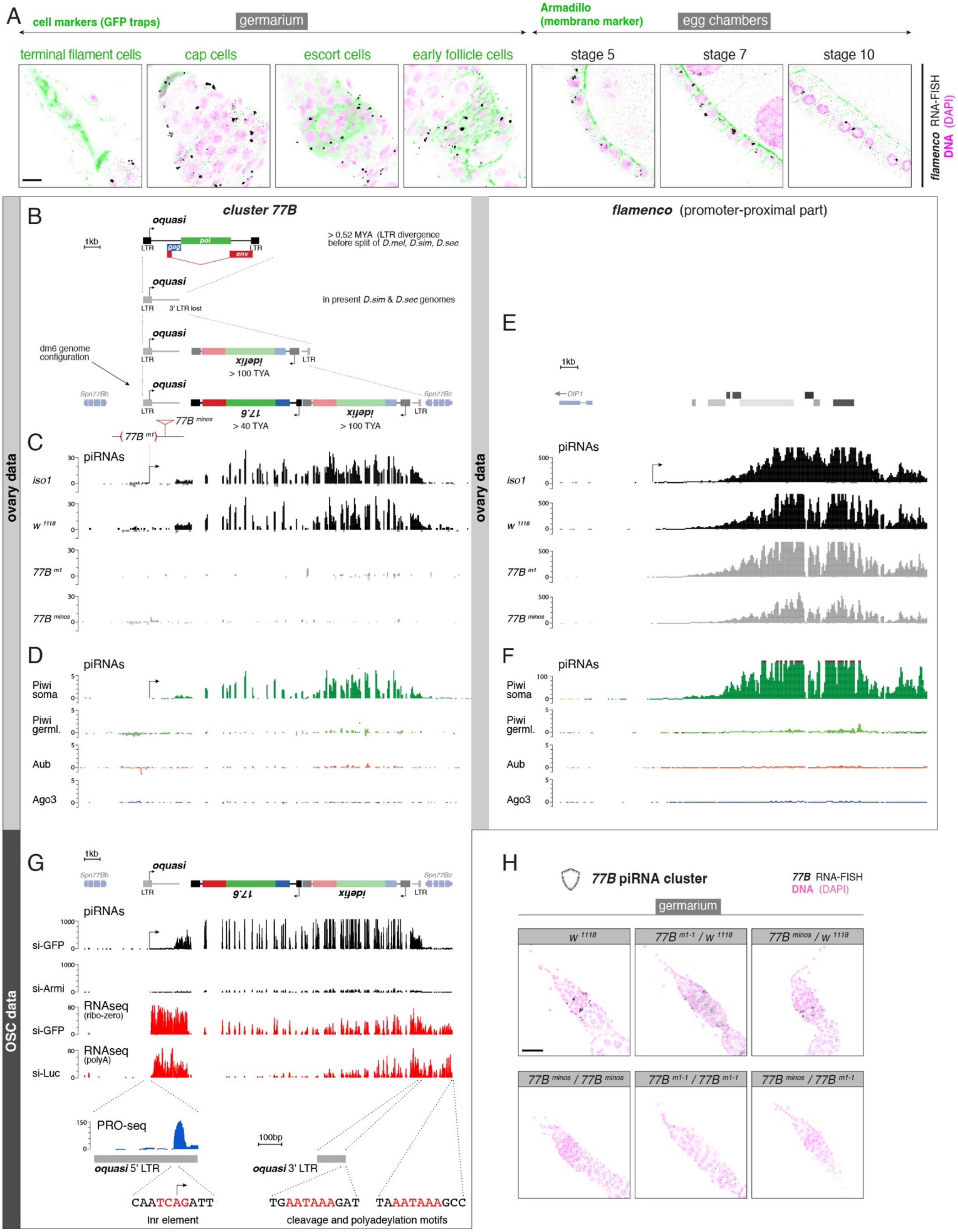

Appendix Figure S17. Characterization of the somatic *77B* piRNA cluster.

A, Detailed RNA smFISH-based expression analysis of the somatic *flamenco* piRNA cluster (black) in ovarian somatic cells (GFP-trap lines in green mark indicated cell types in the germarium; for later stages, an anti-Armadillo stain marks cell outlines; DAPI is shown in magenta, oogenesis stages are indicated; scale bars: 10µm). **B,** Cartoon depicting the proposed evolutionary trajectory of the *77B* piRNA cluster, which originated from an old *quasimodo* retrovirus (*oquasi*) insertion between the *Spn77Bb* and *Spn77Bc* genes and captured an *idefix* and *17.6* retroviral insertion in antisense orientation (scale and alignment of the cartoon corresponds to the data in panels C, D, G). **C, D,** Shown are genome-unique piRNA mappings from ovaries of indicated genotypes (C) or bound to indicated PIWI-clade proteins from wild-type ovaries (D) (Senti *et al.*, 2015), demonstrating that *77B*-derived piRNAs are specifically bound to soma-expressed Piwi (D). **E, F,** As in (C, D), but for the beginning of the *flamenco* piRNA cluster. **G,** Analysis of *cluster 77B* expression in ovarian somatic cells (OSCs), a cell line presumably derived from ovarian follicle stem cells. Shown are genome-unique mappings of piRNA reads, RNA-seq reads (from ribo-zero and from poly A-plus libraries), and PRO-seq reads (all mappers) at the *77B* piRNA cluster. Motifs for transcription initiation (initiator element) and cleavage/poly-adenylation are shown at the bottom. **H,** Expression analysis of the *77B* piRNA cluster in the germarium of ovaries with indicated homozygous and heterozygous genotypes based on RNA-smFISH (black) (DAPI labels nuclei (magenta); images represent maximum intensity projections of ten confocal Z-sections). Scale bar: 20µm.

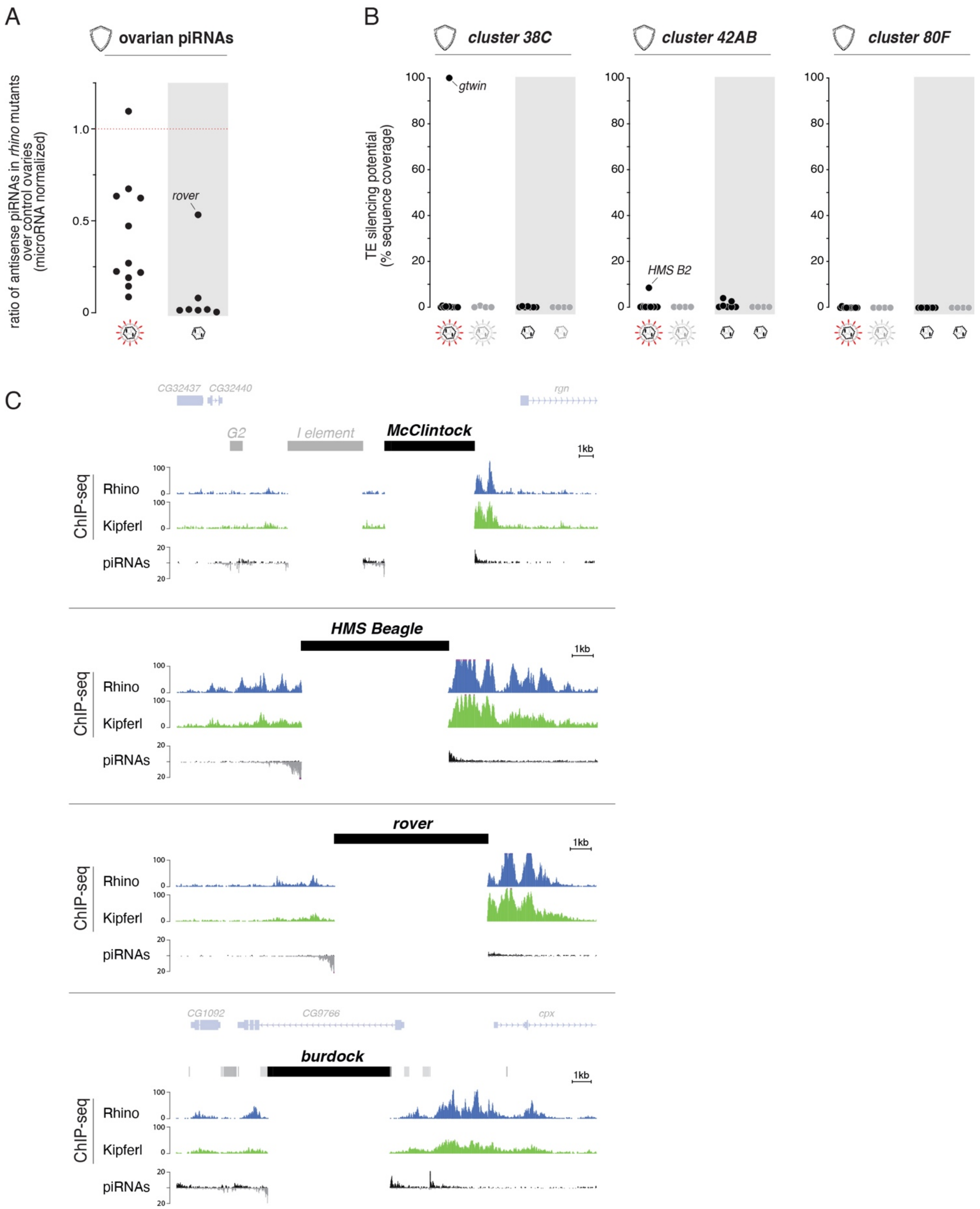

Appendix Figure S18. piRNAs targeting iERV retroelements in the germline originate from stand-alone insertions.

A, Ratios of antisense piRNAs (microRNA normalized) mapping to active retroviruses (left) or retroelements (right) among iERVs are shown from ovaries of germline-specific *rhino* RNAi flies (*MTD*-Gal4 driven shRNA against *rhino*) versus control ovaries (*MTD*-Gal4 driven shRNA against *white*). The transition element *rover* is the

only retroelement with a significant proportion of Rhino-independent piRNAs, consistent with an almost full-length *rover* insertion in *flamenco*. **B,** Shown are the silencing potentials of dual-stranded germline piRNA clusters against active or inactive retroviruses and retroelements among the iERVs. The silencing potentials are expressed as percentage coverage of the TE sequences found within the respective clusters (calculated as 25mers with no mismatches). **C,** Stand-alone insertions of the indicated retroelements acting as mini-piRNA source loci in the germline are shown. Genome-unique reads from Rhino ChIP-seq, Kipferl ChIP-seq and ovarian piRNAs (all from the *iso-1* strain) are shown. Reads mapping to the indicated TE insertions are multi-mapping and not shown.

A

DSPR / *rover*	A1	A2	A3	A4	A5	A6	A7	AB8	B1	B2	B3	B4	B6	iso1	total
LTR408	2	3	1	1	1	3	4	1	1	2	2	3	4	0	28
LTR388	1	0	0	1	0	1	0	0	0	1	1	1	1	1	8
LTR459	1	1	2	1	2	1	1	1	1	1	1	1	1	1	16
LTR315	2	4	1	1	1	1	1	1	1	1	1	1	1	2	19
LTR376	3	1	1	2	2	1	2	3	5	2	1	2	3	4	32
LTR367	3	2	3	5	4	3	0	6	0	3	0	0	2	4	35
total	12	11	8	11	10	10	8	12	8	10	6	8	12	12	

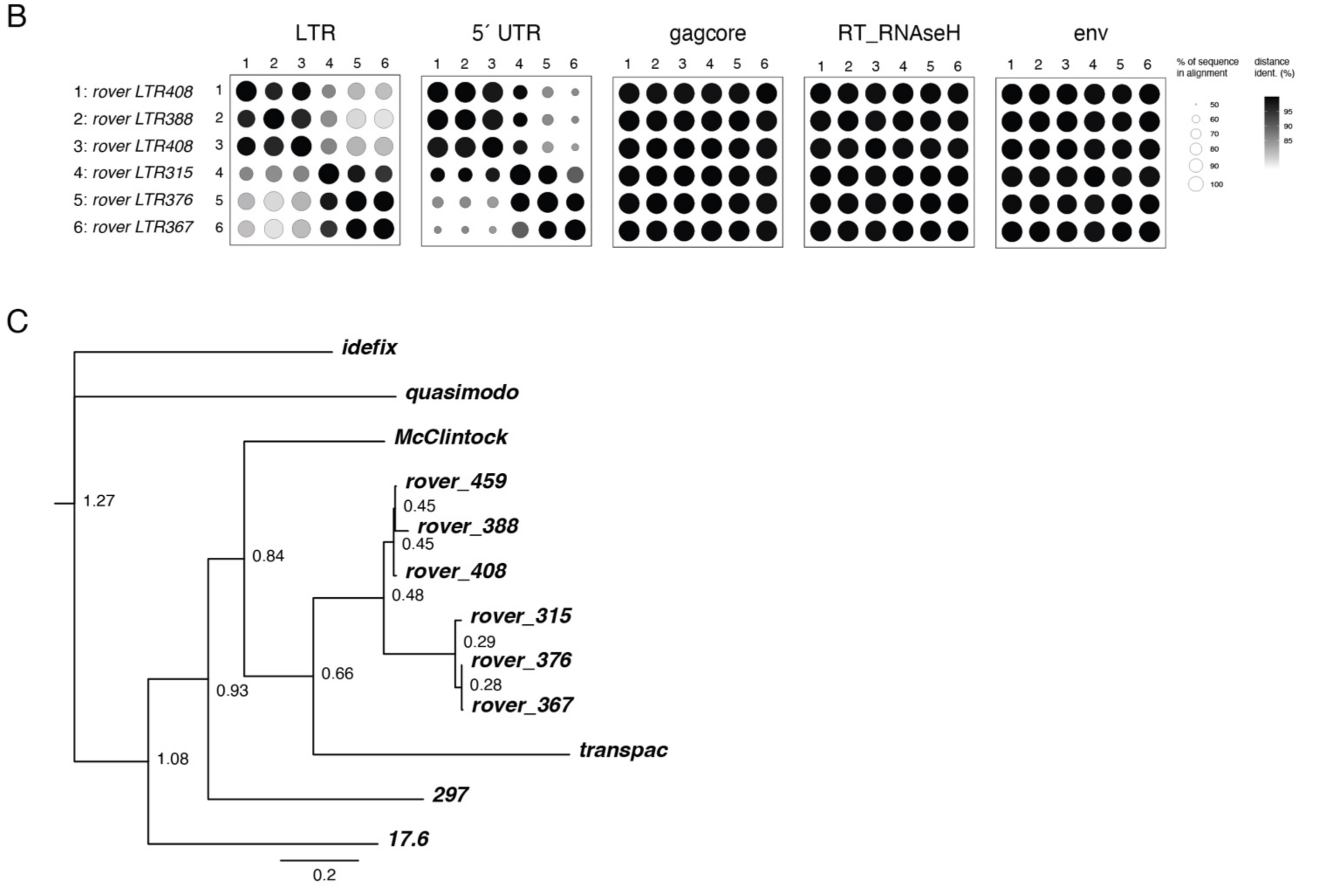

Appendix Figure S19: Analysis of *rover* variant insertion numbers, sequence divergence, LTR phylogeny and insertion ages.

A, Table of analyzed *rover* variant insertions in the DSPR and *iso-1* genomes by type. **B,** Pairwise identities between the DNA sequences of the six representative *rover* variants separately for LTR, 5´ UTR, gag_{core}, $pol_{RT\text{-}RNAseH}$ and *env-F* (as in Figure 4). **C**, Phylogenetic tree (scale indicates nucleotide substitutions per site) based on an alignment of the LTR sequences of the *idefix* subgroup lineages with the LTRs of the six *rover* variants.

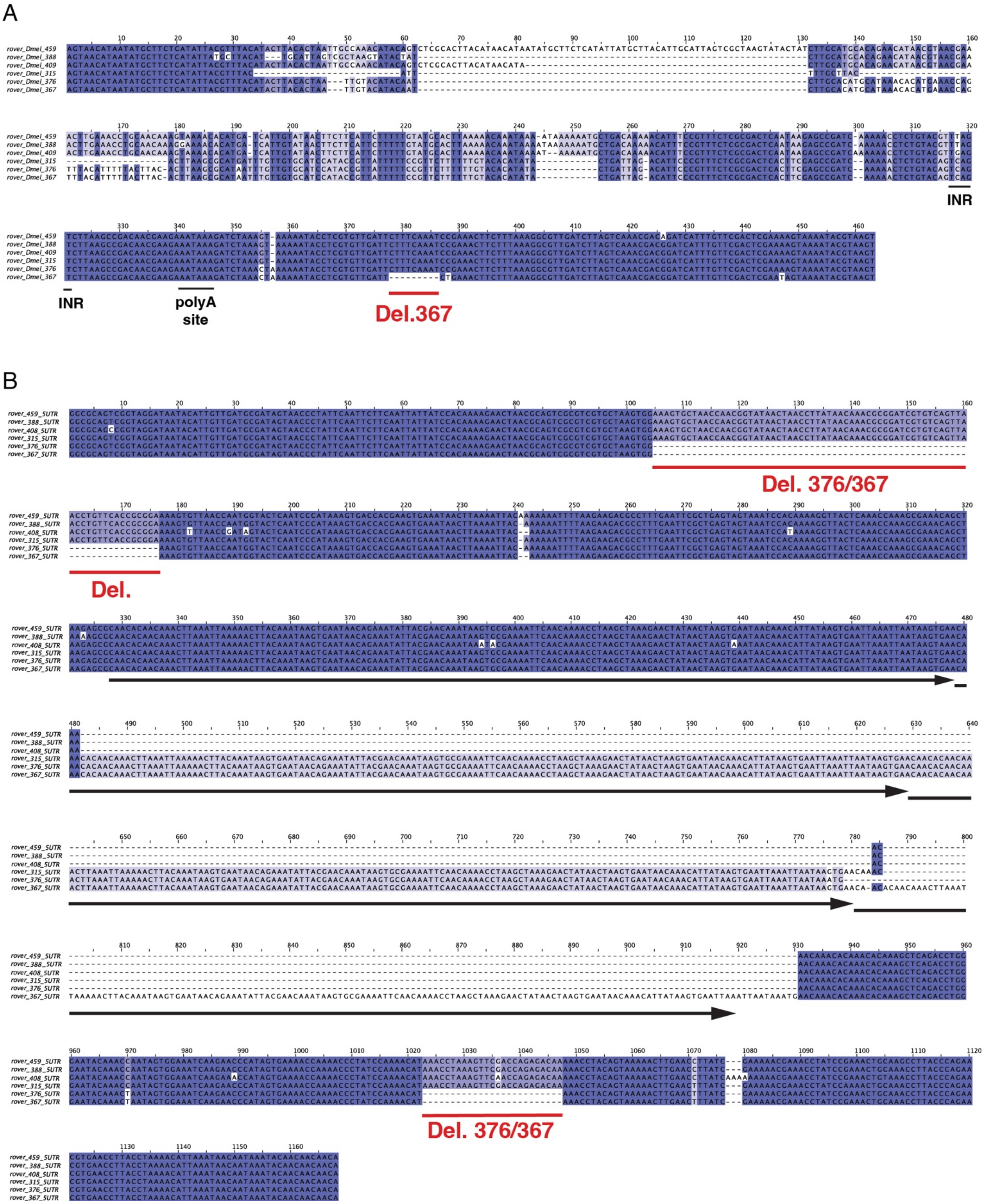

Appendix Figure S20: Sequence divergence of LTRs and 5´ UTRs of six *rover* variants
A, LTR alignment of six respresentative *rover* variant types, LTR-459, LTR-388, LTR-408, LTR-315, LTR-376, LTR-367, with transcriptional start (INR) and stop sites (polyA) indicated. **B,** Corresponding 5´ UTR alignment. Larger *rover* variant deletions are shown in red and variation in the 151bp repeat are indicated with black arrows.

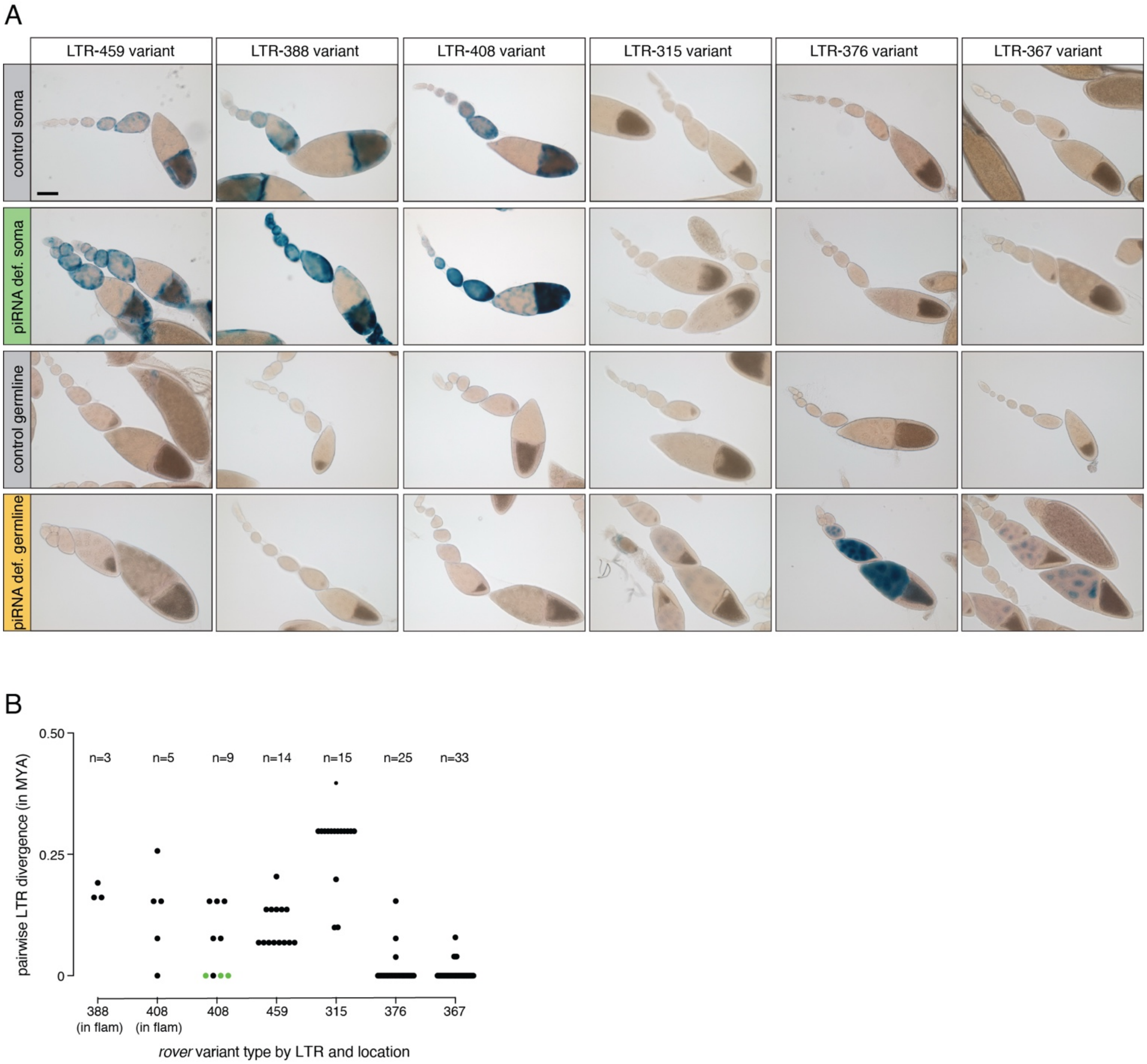

Appendix Figure S21: Transgenic *rover*-lacZ reporter analysis in control and tissue specific piRNA pathway defective flies

A, X-gal staining of ovaries from transgenic flies bearing lacZ reporter constructs using full length *cis*-regulatory sequences of six representative *rover* variants (as in Figure 6). Shown ovaries that are wild type controls or defective for the soma or germline piRNA pathway. Scale bar: 100μm. **D**, Jitter plot showing pairwise LTR divergence-based insertion age estimates in millions of years (MYA) for the *rover* variants inserted in *flamenco* (LTR-388, LTR-408 in *flam*) and those inserted in other genomic locations for (LTR-408, LTR-459, LTR-315, LTR-376, and LTR-367 variants). LTR-408 insertions shown in green represent functionally intact retroviral *rover* variants with no pairwise LTR divergence. We note that some panels in Appendix Figure S21 were intentionally reused from Figure 6E to allow the direct side-by-side comparison of control and genetic depletion of the piRNA pathway.

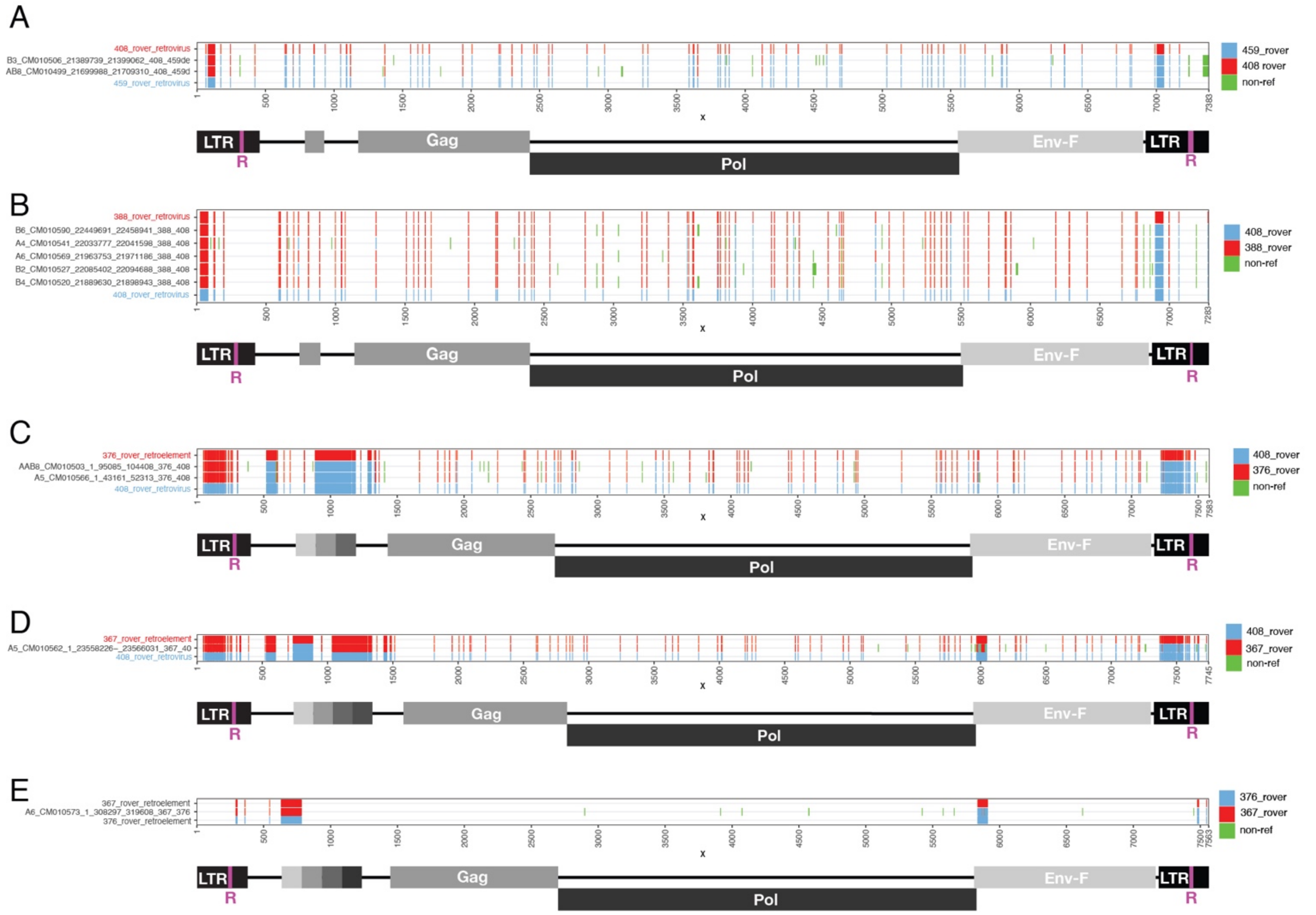

Appendix Figure S22: Analysis of recombinant *rover* insertions with discordant LTRs of different *rover* variants

Sequence variation (SNPs and INDELs) was identified between two typical *rover* non-recombinant variant types (references) and individual identified recombinant *rover* insertions using pairwise sequence comparisons. Sequence variation specific *rover* variant1 are plotted in red and variant 2 in blue. The sequence variation of individual identified recombinant *rover* insertions is shown in red/blue (as for *rover* non-recombinant reference sequences in the alignment) or green (for any non-reference type of variation). Sequence variations are shown along the alignment-based sequence variation plots of the full-length *rover* insertions, with typical *rover* variants shown at the top and bottom and individual identified recombinant insertions plotted between the references. For structural reference to each alignment, *rover* functional sites were mapped to the respective alignment and are displayed as a model below the line plot. **A,** Plot showing a recombination event between the LTR-408 (top) and LTR-459 retroviral *rover* variants (bottom) and two identified insertions with discordant LTRs from the two DSPR strains B3 and AB8 plotted in between. Note the recombinant *rover* insertions show 408bp type LTRs at the 5´end and 459bp type of LTRs in the 3´end. The insertion sites of these recombinant *rover* sequences in B3 and AB8 are identical and located in antisense in *flamenco* and hence likely derive from a single evolutionary recombination event. **B,** Shown is a recombination event between the retroviral LTR-388 (top) and LTR-408 *rover* variants (bottom) and five identified insertions with discordant LTRs of 388bp or 408bp from DSPR strains (A4, A5, B2, B4, and B6) plotted in between. Note the recombinant *rover* insertion lines show 388bp type LTRs at the 5´end and 408bp type of LTRs at the 3´end. The insertion sites of these recombinant *rover* sequences in B6, A4, A5, B2, and B4 are identical and located in antisense in *flamenco* and likely derived from a single evolutionary recombination event. **C,** Shown is a recombination event between the LTR-376 retroelement *rover* variant (top) and the LTR-408 retroviral *rover* variant (bottom) alongside two identified insertions with discordant LTRs of 376bp or 408bp from two DSPR strains (AB8 and A5) plotted in between the two references. Note the recombinant *rover* insertion lines show 376bp type LTRs at the 5´ end and 408bp type of LTRs at the 3´ end. The insertion sites of these recombinant *rover* sequences in A5 and AB8 are identical and located within chr3R heterochromatin, hence likely derive from a single evolutionary recombination event. **D,** Shown is a recombination event between

the LTR-367 retroelement *rover* variant (top) and the LTR-408 retroviral *rover* variant (bottom) and an identified insertion with discordant LTRs of 367bp or 408bp from a single DSPR strain (A5) plotted in between the two references. Note the recombinant *rover* insertion shows a 367bp type LTR at the 5´end and 408bp type of LTR at the 3´end. The insertion site of this recombinant *rover* sequence in A5 is located within chromosome X heterochromatin. **E,** Shown is a recombination event between the two retroelement *rover* variants LTR-367 (top) and LTR-376 variant (bottom) and a single insertion with discordant LTRs of 367bp or 376 bp from a single DSPR strain (A6) in between. Note the recombinant *rover* insertion shows a 367bp type LTR at the 5´end and 376bp type of LTR in the 3´end. The insertion site of this recombinant *rover* sequence in A6 is located within chromosome 3 heterochromatin.

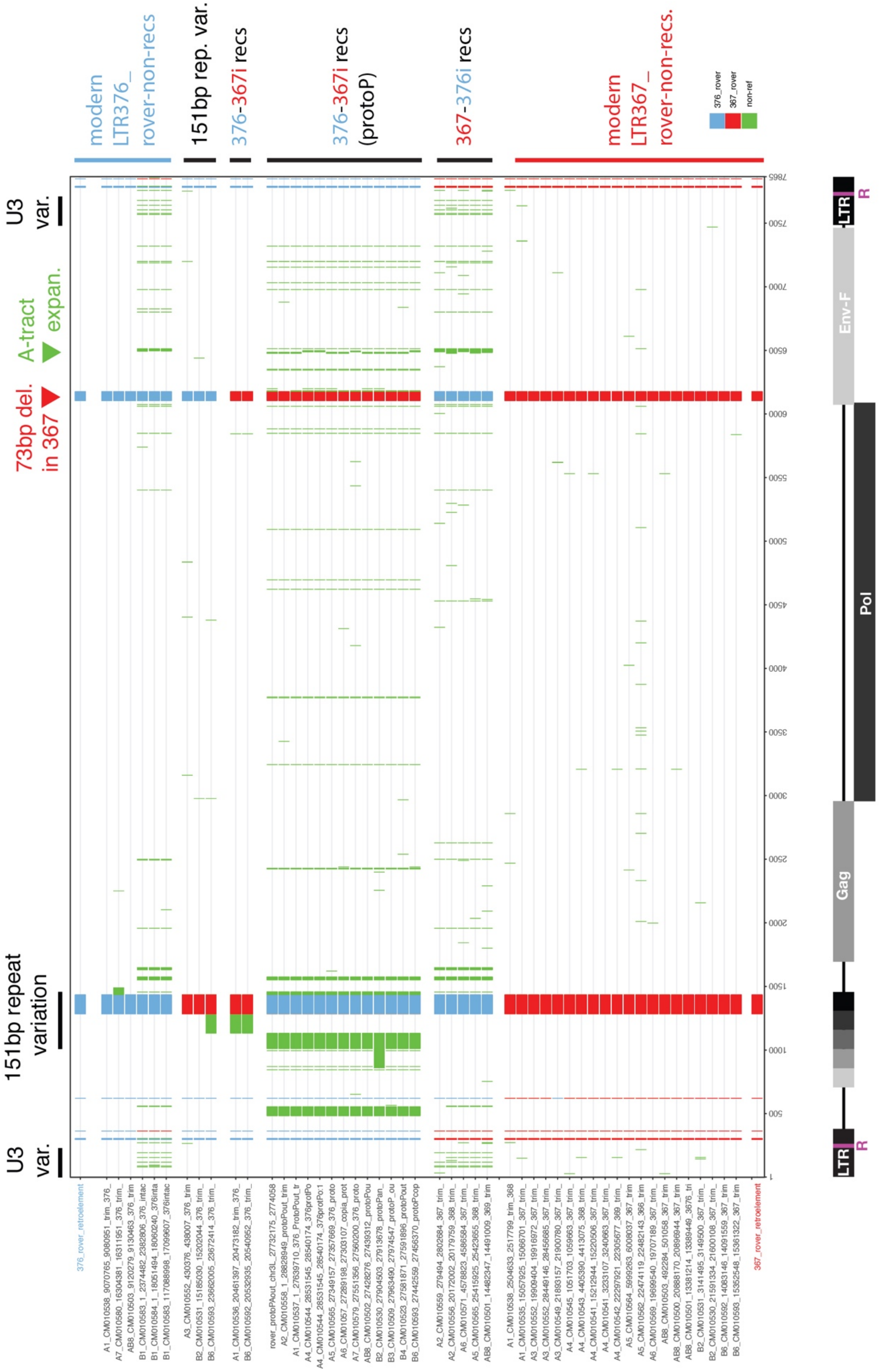

Appendix Figure S23: Internal recombination events between the retroelement LTR-367 and LTR-376 *rover* variants

Recombination analysis (as in Appendix Figure S22) using typical LTR-367 and LTR-376 *rover* retroelements as references (plotted on top and at the bottom) and sequences of 50 insertions of *rover* retroelement variants with concordant LTRs of the 367bp or 376bp types from the DSPR strains and *iso-1*. Most of the LTR-376 insertions are typical none-recombinant type (n=6). Three LTR-376 insertions display copy variation in the 151bp repeat which may derive from or represent reverse transcriptase slippages. Two LTR-376 insertions from two DSPR strains (A1 and B6), both at the same fixed position in chromosome 2R euchromatin and representing a single evolutionary event, have acquired the 73bp deletion in the 5′ end of the *env-F* exon 2 that is characteristic for LTR-367. All thirteen LTR-376 insertions interrupted by a *ProtoP* insertion (fixed in most analyzed strains and therefore representing a single recombination event) also show the LTR-367 type 73bp deletion in *env-F*. Most LTR-367 insertions do not display recombination events (n=20). Five LTR-367 insertions have lost the 73bp deletion at the 5′ end of *env-F* exon 2 that is characteristic for the LTR-367 variant in exchange for the sequence characteristic for LTR-376. The same five LTR-367 insertions show also a reduced copy number of the 151bp repeat in the 5′ UTR from four to three repeats.

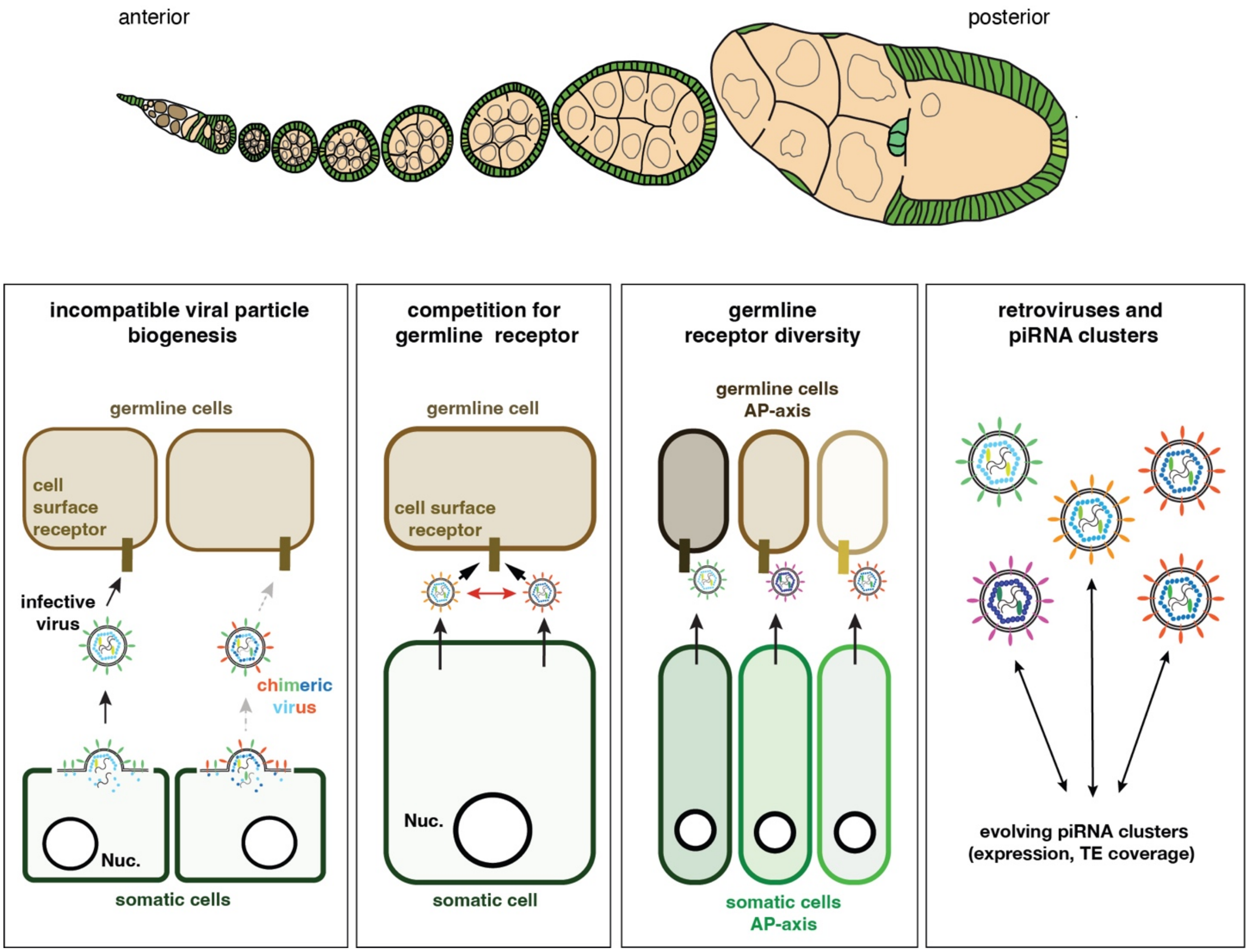

Appendix Figure S24. Possible scenarios of competition between infectious iERVs and between iERVs and the *Drosophila* host.
