# Supplementary material for: Co-evolving infectivity and expression patterns drive the diversification of endogenous retroviruses
Source: EMBO J. 2025 Jun 5;45(6):1889–908. doi: 10.1038/s44318-025-00471-8 (PMC12992720; doi:10.1038/s44318-025-00471-8)
Supplement: Supplementary file 3 — Dataset EV1 [file 44318_2025_471_MOESM3_ESM.zip › EV1 dataset readme.rtf]

EV1 dataset: Detailed experimental reagents and tools used in this studysheet 1: Primer sequences used to amplify the spliced Env-F transcripts of infective iERVs from ovarian RNA.sheet 2: Drosophila melanogaster strains usedsheet 3: Compiled single molecule fluorescent in situ hybridisation (smFISH) oligo sequencessheet 4: Input reagents (template DNA) and primers used to construct the transcriptional iERV reporters sheet 5: piRNA cluster coordinates in the dm6 reference genome usedsheet 6: List of all polyA-RNA-seq libraries generated and analyzed
