# Supplementary material for: Co-evolving infectivity and expression patterns drive the diversification of endogenous retroviruses
Source: EMBO J. 2025 Jun 5;45(6):1889–908. doi: 10.1038/s44318-025-00471-8 (PMC12992720; doi:10.1038/s44318-025-00471-8)
Supplement: Supplementary file 4 — Dataset EV2 [file 44318_2025_471_MOESM4_ESM.zip › EV2 dataset readme.rtf]

EV2 dataset: Curated consensus sequences of insect endogenous retroviruses in EMBL sequence formatEach consensus sequence was annotated for LTRs, Open Reading Frames of Gag, Pol and the two exons of the spliced envelope-F (Env-F) from previously available (BDGP or Repbase) or from our experimental data. The appendix “_cur” was added to indicate our revised sequence annotations.The list contains the following sequences: gypsy5, ZAM, tirant, accord, accord2, idefix, quasimodo, rover, McClintock, 297, 17.6, transpac, chouto, gypsy4, gypsy10, gypsy9, burdock, gypsy7, gypsy, gtwin, gypsy2, gypsy6, springer, gypsy3, HMS-Beagle, HMS-Beagle2, and opus.
